# Using UHPLC-MS Profiling for the Discovery of New Dihydro-β-Agarofurans from Australian Celastraceae Plant Extracts

**DOI:** 10.3390/molecules24050859

**Published:** 2019-02-28

**Authors:** Mario Wibowo, Paul I. Forster, Gordon P. Guymer, Andreas Hofmann, Rohan A. Davis

**Affiliations:** 1 Griffith Institute for Drug Discovery, Griffith University, Brisbane, QLD 4111, Australia; mario.wibowo@griffithuni.edu.au (M.W.); a.hofmann@griffith.edu.au (A.H.); 2 Queensland Herbarium, Brisbane Botanic Gardens, Toowong, QLD 4066, Australia; paul.forster@des.qld.gov.au (P.I.F.); gordon.guymer@des.qld.gov.au (G.P.G.); 3 Department of Veterinary Biosciences, Melbourne Veterinary School, The University of Melbourne, Melbourne, VIC 3010, Australia

**Keywords:** UHPLC-MS, Celastraceae, dihydro-β-agarofuran, sesquiterpenoid, *Denhamia celastroides*, NMR

## Abstract

An analytical method using UHPLC-MS was developed and applied to 16 crude CH_2_Cl_2_ extracts from Australian Celastraceae plants; the endemic plant materials were accessed from Griffith University’s NatureBank resource and included bark, fruit, leaf, root, twig and mixed samples, all of which were collected from Queensland, Australia. The generated UHPLC-MS data were analysed and dereplicated using the scientific databases Dictionary of Natural Products and SciFinder Scholar in order to potentially identify new dihydro-β-agarofurans from local Celastraceae plants. These investigations led to the large-scale extraction and isolation work on a prioritised fruit sample that belonged to the rainforest plant *Denhamia celastroides*. Chemical investigations resulted in the purification of four new natural products, denhaminols O–R (**1**–**4**), along with the related and known compound, denhaminol G (**5**). The structures of all the new compounds were determined via detailed analysis of NMR and MS data.

## 1. Introduction

Dihydro-β-agarofurans are a class of structurally unique polyoxygenated tricyclic sesquiterpenoids, which incorporate a *trans*-decalin and a tetrahydrofuran and are commonly found in the Celastraceae plant family [1]. This class of natural products has gained much attention due to their various and promising bioactivities, such as multidrug resistance reversal [2], antitumor-promotion [3], acetylcholinesterase inhibition [4], antifungal [5], *α*-glucosidase inhibition [6], antiplasmodial [7] and leucine uptake inhibition [8]. The biological effects of dihydro-β-agarofurans are related not only to several stereocentres but also to acyl groups attached to the tricyclic core scaffold. One of our group’s current research interests is the isolation of new natural products (i.e., dihydro-β-agarofurans) from Australian Celastraceae native plants [8,9,10].

Within the workflow of natural product research, the time-consuming re-isolation of previously identified compounds presents a major obstacle and can significantly delay discovery efforts [11]. However, rapid and detailed dereplication methodologies can solve this problem. In the context of our continuing interest in the identification of new natural products from Celastraceae plants [8,9,10], we sought to establish a new UHPLC-MS dereplication method that can guide prioritisation of biota samples and expedite the discovery of new secondary metabolites.

UHPLC-MS is becoming an important tool in natural product dereplication as it allows for fast fingerprinting and profiling analysis [12,13,14]. Moreover, the use of MS in conjunction with the UHPLC system also provides key structural information, such as molecular weight and diagnostic fragments [12,15,16]. Therefore, in the current work we utilised UHPLC-MS and scientific database (Dictionary of Natural Products [17] and SciFinder Scholar [18]) analysis to rapidly undertake dereplication and prioritise Australian Celastraceae plant samples for detailed chemical investigation work, with the ultimate goal of identifying new dihydro-β-agarofurans. The Celastraceae plants used for these studies were all accessed from NatureBank, which is a unique biodiscovery resource based on natural products derived from Australian plants, fungi and marine invertebrates. This facility is located at the Griffith Institute for Drug Discovery (Griffith University) and currently holds an 18,000 extract library, a 90,000 fraction library and >30,000 archived biota samples [19]. The 16 NatureBank plant samples used in these particular studies included three bark, one fruit, one leaf, seven root, two twig and two mixed samples collected from Queensland, Australia. The CH_2_Cl_2_ extracts of all 16 plant samples were subjected to UHPLC-MS. A total of three samples were prioritised for potential large-scale extraction and isolation studies; specifically, the selected samples included the fruits of *Denhamia celastroides* since UHPLC-MS data in conjunction with database analyses suggested the presence of new dihydro-β-agarofurans. Large-scale extraction and MS-guided isolation of the fruits of *D. celastroides* led to the discovery of four previously undescribed dihydro-β-agarofurans (denhaminols O–R, **1**–**4**) and a known congener denhaminol G (**5**). The structures of all new compounds were assigned by 1D/2D NMR and MS data analysis.

This report describes a simple and rapid method for the generation of Celastraceae plant extracts and subsequent UHPLC-MS analysis, dereplication and prioritisation that has successfully led to the identification of four new plant secondary metabolites.

## 2. Results and Discussion

**Figure 1:**
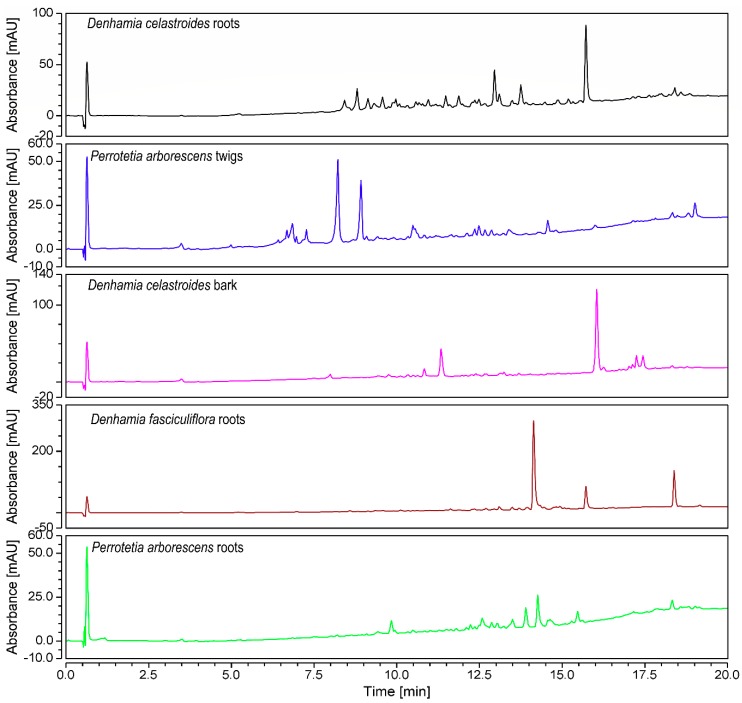
Five representative UHPLC chromatograms (254 nm) of the 16 Celastraceae plants CH_2_Cl_2_ extracts (for all chromatograms see Figure S1, Supplementary data).

**Figure 2:**
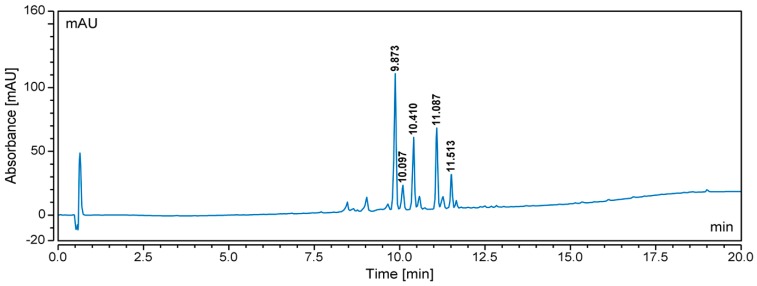
UHPLC chromatogram (254 nm) of the CH_2_Cl_2_ extract of *D. celastroides* fruits; retention times for major UV peaks are indicated.

The CH_2_Cl_2_ extracts of samples from 16 Australian Celastraceae plants were prepared using a small amount of the air-dried and ground samples. All extracts were subjected to UHPLC-MS profiling (Figure 1 and Figure S1, Supplementary data) and the molecular masses of the major UV-active compounds in each of the extracts were determined from either the negative or positive total ion chromatogram (TIC). These data were then analysed using SciFinder Scholar and the Dictionary of Natural Products (DNP). Examination of respective UHPLC chromatograms and scientific databases provided a preliminary overview of the constituents of the extracts. The number of hits (from the database search) of the molecular weights generated from the MS data was used as a filter. Of the 16 plant extracts subjected to UHPLC-MS, three plant samples [*Denhamia celastroides* (F. Muell.) Jessup - fruits, *Hysophila halleyana* (F. Muell.) - bark and *Perrottetia arborescens* (F. Muell.) Loes - root] were prioritised since distinct molecular ions were detected (less than five hits reported in the databases; Table S2, Supplementary data). Since the UHPLC traces of the fruit extract of *D. celastroides* showed the best separation amongst all analysed plant extracts (Figure 2), this sample was chosen for additional chemical investigations.

The scientific databases search (Table 1 and Table S2, Supplementary data) indicated the possibility of new dihydro-β-agarofurans present in the fruits of a *Denhamia celastroides* CH_2_Cl_2_ extract since it contained several distinct molecular ions in the (+)-ESI mode (*m*/*z* 615 [M + H]^+^; 631 [M + H]^+^; and 657 [M + H]^+^), which were only found to match a few dihydro-β-agarofurans (less than five hits) reported in SciFinder Scholar and DNP search (keywords: “Celastraceae” and “agarofuran”). To confirm the presence of the new compounds and to unambiguously identify the structures, we conducted a large-scale extraction of the plant material and MS-directed purification to isolate the targeted compounds with the distinct molecular weights before NMR experiments were conducted.

**Figure 3:**
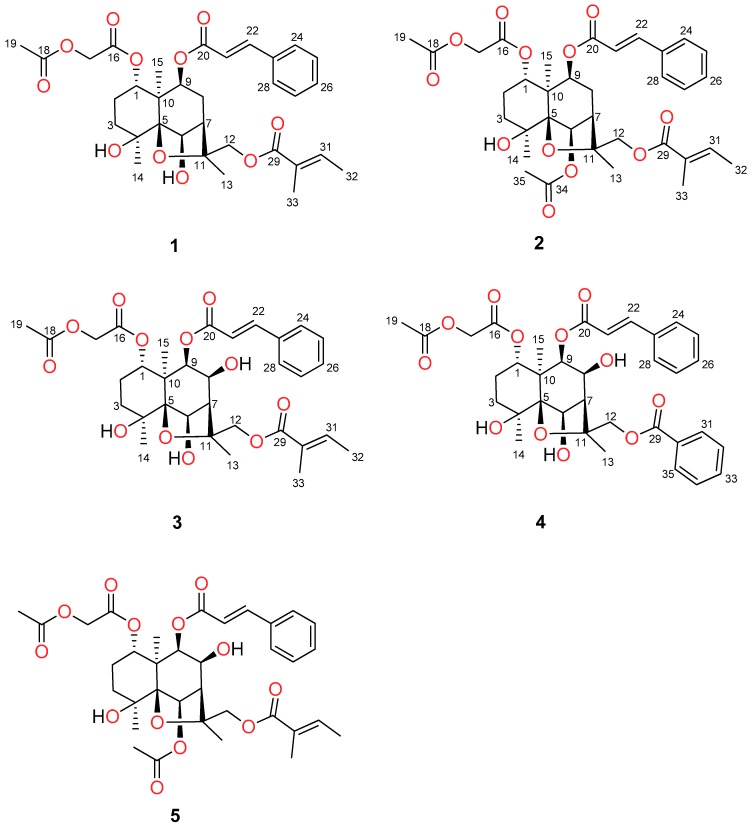
Chemical structures of denhaminols O–R (**1**–**4**) and denhaminol G (**5**).

The fruits of *D. celastroides* (10 g) were sequentially extracted with CH_2_Cl_2_. Subsequent purifications using silica gel column chromatography and RP-HPLC afforded five dihydro-β-agarofurans (Figure 3). The targeted compounds with molecular ions *m*/*z* [M + H]^+^ of 615, 631 and 657 were confirmed to be new natural products, which were given the trivial name denhaminols O–Q (**1**–**3**), respectively. During the isolation of the three targeted compounds, another new dihydro-β-agarofuran (denhaminol R, **4**) and a known congener, denhaminol G (**5**) [20] were also obtained. The complete structure elucidation of the new compounds is detailed below.

**Figure 4:**
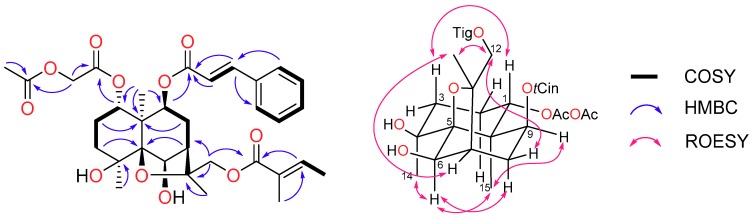
Diagnostic 2D NMR correlations for denhaminol O (**1**).

Denhaminol O (**1**) was isolated as a colourless gum with a molecular formula of C_33_H_42_O_11_ as assigned by HRESIMS data (*m*/*z* 637.2595). The ^1^H-NMR spectrum (Table 2) exhibited signals of 6 methyl protons (δ_H_ 1.33, 1.59, 1.63, 1.78, 1.79 and 1.88), 5 methylene protons (δ_H_ 1.53/1.95, 1.69/1.86, 2.03/2.26, 4.31/4.45 and 4.52/4.69) and 12 methine protons (δ_H_ 2.37, 4.44, 4.88, 5.39, 6.40, 6.81, 7.36 (3H), 7.56 (2H) and 7.65). The ^13^C (Table 3) and HSQC spectra of **1** suggested a total of 33 carbons, including 6 methyls, 5 methylenes, 12 methines and 10 non-protonated carbons. The ^13^C resonances at δ_C_ 165.9, 167.1, 167.6 and 170.2 suggested the presence of four ester groups in **1**. These data indicated that compound **1** was a dihydro-β-agarofuran bearing four ester groups [8,9,10], which was confirmed by COSY and HMBC experiments (Figure 4).

The positions of the ester groups were determined following HMBC data analysis. HMBC cross-peaks from two olefinic protons (δ_H_ 6.40 and 7.65, d, *J* = 16.0) and H-9 (δ_H_ 4.88) to an ester carbonyl carbon at δ_H_ 165.9 located a *trans*-cinnamate group at C-9. The ^1^H-NMR resonances at δ_H_ 1.78 (3H, m), 1.79 (3H, m) and 6.81 (1H, m) were the characteristic of tigloyl moiety [20]. The tigloyl group was located at C-12 based on HMBC correlations from a pair of diastereotopic methylene protons (δ_H_ 4.52 and 4.69), a methyl at δ_H_ 1.79 and an olefinic proton at δ_H_ 6.81 to a carbonyl resonance at δ_C_ 167.6. The HMBC spectrum of **1** also exhibited correlations from a set of methylene protons at δ_H_ 4.31 and 4.45 to two carbonyl carbons resonating at δ_C_ 167.1 and 170.2. These data along with further HMBC correlation from H-1 (δ_H_ 4.88) to the carbonyl carbon at 167.1 suggested the location of an acetoxyacetate functional group at C-1. Finally, two hydroxy moieties were positioned at C-4 and C-6 by considering the molecular formula of **1** and the deshielded NMR resonances of C-4 (δ_C_ 73.3) and CH-6 (δ_H_ 4.44 and δ_C_ 79.4). The relative configuration of denhaminol O (**1**) was established by ROESY (Figure 4) and ^1^H-^1^H coupling constant data analysis. The large coupling constant of H-1 (*J*_1,2_ = 12.1 Hz) indicated the β-orientation of H-1. Similarly, the *α*-orientation of H-9 was assigned based on the coupling constant (*J*_8,9_ = 7.4 Hz). ROESY cross-peaks between H_3_-14 and H-6 and between H-6 and H-9 as well as between H-9 and H_3_-15 suggested that these protons were cofacial. It is worth mentioning that ROESY correlations were also observed between H_2_-12 and H-8β as well as between H_3_-13 and H-7. Consequently, the structure of **1** was established as 1*α*-acetoxyacetate-8β-cinnamoyloxy-4β,6β-dihydroxy-12-tigloyloxydihydro-β-agarofuran.

Denhaminol P (**2**, colourless gum) had a molecular formula of C_35_H_44_O_12_ as assigned by (+)-HRESIMS. Comparison of ^1^H and ^13^C-NMR spectra of **2** and **1** showed a high degree of similarity between the two compounds. However, the ^1^H resonance of H-6 was shifted downfield from δ_H_ 4.44 in **1** to δ_H_ 5.48 in **2**, which suggested the attachment of an ester group at C-6 in **2**. HMBC correlations from a methyl singlet signal at δ_H_ 2.15 and H-6 (δ_H_ 5.48) to a carbonyl carbon at δ_C_ 170.6 identified the presence of an acetate group at C-6 in **2**. The 2D NMR data (Figure S28, Supplementary data) further confirmed the attachment of the remaining ester moieties in **2**. Comparison of ROESY data of **1** and **2** revealed the same relative configurations for these compounds. Thus, the structure of **2** was elucidated as 6β-acetoxy-1*α*-acetoxyacetate-8β-cinnamoyloxy-4β-hydroxy-12-tigloyloxydihydro-β-agarofuran.

Compound **3** was isolated as a colourless gum and had a molecular formula of C_33_H_42_O_12_ as suggested by HRESIMS. The NMR data of **3** were similar to those of **1**, except for the presence of an additional hydroxy in **3**. The hydroxy group was positioned at C-8 based on the deshielded resonances of CH-8 (δ_H_ 4.30 and δ_C_ 70.3). HMBC correlations (Figure S29, Supplementary data) from H-8 to C-6, C-9 and C-11 further confirmed the location of OH-8. The β-orientation of the hydroxy moiety at C-8 was assigned by ROESY correlations (Figure S29, Supplementary data) between H-8 and H_3_-15. Therefore, the structure of denhaminol Q (**3**) was determined as 1*α*-acetoxyacetate-8β-cinnamoyloxy-4β,6β,8β-trihydroxy-12-tigloyloxydihydro-β-agarofuran.

During the isolation of the major compounds **1**–**3**, denhaminol R (**4**) was obtained. The molecular formula of compound **4** was C_35_H_40_O_12_ as indicated by (+)-HRESIMS data. Analysis of NMR and MS data showed that the structure of **4** was similar to that of **3**. However, the tigloyl group at C-12 in **3** was replaced by a benzoate in **4**. The presence of the benzoate group was confirmed by the characteristic ^1^H NMR resonances at δ_H_ 7.45 (2H, m), 7.57 (1H, m) and 8.03 (2H, m). The position of the benzoate at C-12 was assigned by HMBC correlations (Figure S30, Supplementary data) from H_2_-12 (δ_H_ 4.88 and 5.02) and the proton at δ_H_ 8.03 to an ester carbonyl carbon at δ_C_ 166.4. The relative configurations of **4** were ascertained to be the same as those of **3** by ROESY experiment. Accordingly, the structure compound **4** was assigned as 1*α*-acetoxyacetate-12-benzoylyloxy-8β-cinnamoyloxy-4β,6β,8β-trihydroxydihydro-β-agarofuran.

Previously, we reported the isolation of dihydro-β-agarofuran sesquiterpenoids from two Australian plants belonging to the *Denhamia* genus, namely *D. celastroides* and *D. pittosporoides*. The chemical investigation of the leaves of *D. celastroides* afforded eight dihydro-β-agarofurans (denhaminol A–H) [20], while two new dihydro-β-agarofurans (denhaminols I and J) were obtained from the leaves of *D. pittosporoides* [9].

## 3. Materials and Methods

### 3.1. General Experimental Procedures

Values of specific rotations were determined with a JASCO P-1020 polarimeter and UV spectra were recorded using a JASCO V-650 UV/vis spectrophotometer (ATA Scientific, Taren Point, NSW, Australia). ECD spectra were obtained on a JASCO J-715 spectropolarimeter (Tokyo, Japan) and processed using the software SDAR v3.2 [21]. IR data were acquired using an attached universal attenuated total reflectance (UATR) two module on a PerkinElmer spectrophotometer (Waltham, MA, USA). NMR spectra were acquired from a Bruker AVANCE HDX 800 MHz NMR spectrometer (Zurich, Switzerland) equipped with a TCI cryoprobe at 25 °C. The ^1^H and ^13^C chemical shifts were referenced to the residual solvent signal of CDCl_3_ at δ_H_ 7.26 and δ_C_ 77.16 ppm, respectively. HRESIMS data were acquired on a Bruker maXis II ETD ESI-qTOF (Bremen, Germany) and the mass spectrum was calibrated externally with 0.1 mg/mL of sodium trifluoroacetate. A Fritsch Universal Cutting Mill Pulverisette 19 (Idar-Oberstein, Germany) was used to grind the air-dried plant material and an Edwards Instrument Company Bio-line orbital shaker (Narangba, Australia) was used for plant extraction. Phenomenex Strata solid phase extraction (SPE) cartridges (3 cc, polypropylene, single fritted, catalogue# AH0-7806) (Torrance, California, USA) were used for the small-scale plant extractions. The UHPLC-MS was performed on an Ultimate 3000 RS UHPLC (Waltham, MA, USA) coupled to a Thermo Fisher Scientific MSQ Plus single quadruple ESI mass spectrometer (Waltham, MA, USA) using an analytical Waters ACQUITY UPLC CSH C_18_ column (2.1 × 50 mm, 1.7 µm, 130 Å) (Milford, MA, USA). A Thermo Fisher Scientific Dionex Ultimate 3000 UHPLC was used for semi-preparative HPLC separations. A Phenomenex Luna C_18_ (250 × 10 mm, 5 μm, 90–110 Å) column (Torrance, CA, USA) was used for semi-preparative HPLC separations. Alltech C_18_-bonded Si (35–75 μm, 150 Å) (Sydney, NSW, Australia) was used for pre-adsorption work, and the resulting material was packed into an Alltech stainless steel guard cartridge (10 × 30 mm) prior to semi-preparative HPLC separations. Merck Si gel (0.040–0.063 mm, 230–400 mesh) (Darmstadt, Germany) was used for Si gel column chromatography. All solvents (CH_2_Cl_2_ and CH_3_CN) used for chromatography, specific rotation, ECD, UV and MS were RCI Labscan HPLC grade (Samutsakhorn, Thailand). H_2_O was Sartorius arium pro VF (Göttingen, Germany) filtered. All compounds were analysed for purity by ^1^H NMR spectroscopy and shown to be >95%, unless otherwise stated. NMR spectra were processed using MestReNova version 11.0 (Santiago de Compostela, Spain).

### 3.2. Plant Materials

The 16 Celastraceae plant samples were obtained from the NatureBank biota library housed at the Griffith Institute for Drug Discovery, Griffith University, Australia [19]. All samples were collected in Queensland and taxonomically identified by the Queensland Herbarium. Voucher specimens have been deposited at the Queensland Herbarium, Australia. All plant specimens were air-dried, ground and stored at room temperature prior to extraction. The fruits of *Denhamia celastroides* (F. Muell.) Jessup (Voucher specimen code: AQ605014) that were used in the large-scale extraction and isolation investigations were collected on 26 November 1997 in Mt Windsor Tableland rainforest, Queensland, Australia. Details of the collection date, location and voucher specimen codes for the other Celastraceae plants used in these studies are provided in Table S31 of the supplementary data.

### 3.3. Preparation of Crude Plant Extracts for UHPLC-MS Analyses

Each of the air-dried and ground Celastraceae plant materials (300 mg) was packed into an SPE cartridge and extracted under gravity with 8 mL of CH_2_Cl_2_. The CH_2_Cl_2_ extracts were dried, weighed and resuspended in CH_3_CN to generate a stock solution, which had a concentration of 1 mg/mL (minimum stock solution volume = 0.5 mL).

### 3.4. UHPLC-MS Conditions

All CH_2_Cl_2_ extracts were subjected to UHPLC-MS analysis (5 µL injection volume). UHPLC-MS experiments were performed with an Ultimate 3000 RS UHPLC coupled to a Thermo Fisher MSQ Plus single quadruple ESI mass spectrometer (Waltham, MA, USA) using an analytical Waters ACQUITY UPLC CSH C_18_ column (2.1 × 50 mm, 1.7 µm, 130 Å) (Milford, MA, USA). Employing a flowrate of 0.3 mL/min, a gradient of 10% CH_3_CN (0.1% formic acid) in H_2_O (0.1% formic acid) to 100% CH_3_CN (0.1% formic acid) was applied over 15 min, followed by isocratic elution of CH_3_CN (0.1% formic acid).

### 3.5. Large-scale Extraction and Isolation of the Fruits of D. celastroides

The air-dried and ground fruits of *D. celastroides* (10 g) were extracted with CH_2_Cl_2_ (2 × 500 mL for 16 h each) to afford 245.8 mg of a crude extract. The extract was subjected to chromatography using a Si-gel column (3 × 8 cm) and a step-wise gradient system of *n*-hexane/EtOAc (100% *n*–hexane to 100% EtOAc, 10% increment, 100 mL each) to afford 11 fractions (fractions 1–11). All 11 fractions were analysed by UPLC-MS and fractions 7–9 were chosen for further purification based on UPLC-MS data analysis. Fraction 7 (22.4 mg) was pre-adsorbed to C_18_ bonded Si-gel (~1 g), packed into a guard cartridge and attached to a semi-preparative C_18_ HPLC column. A linear gradient from 45% CH_3_CN/H_2_O to 90% CH_3_CN/H_2_O at a flowrate of 4 mL/min was run over 60 min to obtain denhaminol O (**1**, 10.4 mg, *t*_R_ 18–19 min, 0.104% dry *wt*) and denhaminol Q (**2**, 1.5 mg, *t*_R_ 21 min, 0.015% dry *wt*). Fraction 8 (38.4 mg) was pre-adsorbed to C_18_ bonded Si-gel (~1 g), packed into a guard cartridge and attached to a semi-preparative C_18_ HPLC column. A linear gradient from 45% CH_3_CN/H_2_O to 80% CH_3_CN/H_2_O at a flowrate of 4 mL/min was run over 60 min to afford the known natural product, denhaminol G (**5**, 13.9 mg, *t*_R_ 24 min, 0.139 % dry *wt*). Fraction 9 (29.4 mg) was pre-adsorbed to C_18_ bonded Si-gel (~1 g), packed into a guard cartridge and attached to a semi-preparative C_18_ HPLC column. A linear gradient from 40% CH_3_CN/H_2_O to 70% CH_3_CN/H_2_O at a flowrate of 4 mL/min was run over 60 min to yield denhaminol P (**3**, 10.2 mg, *t*_R_ 27–28 min, 0.102 % dry *wt*) and denhaminol R (**4**, 2.2 mg, *t*_R_ 29–30 min, 0.022 % dry *wt*).

### 3.6. Denhaminol O (**1**)

Colourless gum; ${\left[\alpha \right]}_{D}^{24}$–40.2 (*c* 0.520, MeOH); ECD *λ*_ext_ (MeOH) 214 (−4.63), 231 (0.19), 267 (−3.55) nm; UV (MeOH) *λ*_max_ (log *ε*) 281 (4.36) nm; IR (UATR) *ν*_max_ 3409, 2979, 1747, 1705, 1634, 1386, 1256, 1197, 1161, 1077, 973, 768 cm^−1^; ^1^H-NMR (CDCl_3_, 800 MHz) see Table 2; ^13^C-NMR (CDCl_3_, 200 MHz) see Table 3; (+)-LRESIMS *m*/*z* 615 [M + H]^+^; (+)-HRESIMS *m*/*z* 637.2595 [M + Na]^+^ (calcd for C_33_H_42_O_11_Na, 637.2619).

### 3.7. Denhaminol P (**2**)

Colourless gum; ${\left[\alpha \right]}_{D}^{24}$–36.0 (*c* 0.075, MeOH); ECD *λ*_ext_ (MeOH) 217 (−4.79), 268 (−3.81) nm; UV (MeOH) *λ*_max_ (log *ε*) 282 (4.38) nm; IR (UATR) *ν*_max_ 2952, 1739, 1705, 1373, 1197, 1162, 1076, 975, 769 cm^−1^; ^1^H-NMR (CDCl_3_, 800 MHz) see Table 2; ^13^C-NMR (CDCl_3_, 200 MHz) see Table 3; (+)-LRESIMS *m*/*z* 657 [M + H]^+^; (+)-HRESIMS *m*/*z* 679.2693 [M + Na]^+^ (calcd for C_35_H_44_O_12_Na, 679.2725).

### 3.8. Denhaminol Q (**3**)

Colourless gum; ${\left[\alpha \right]}_{D}^{24}$–33.3 (*c* 0.510, MeOH); ECD *λ*_ext_ (MeOH) 216 (−4.60), 253 (−2.01), 301 (0.29) nm; UV (MeOH) *λ*_max_ (log *ε*) 630 (4.441) nm; IR (UATR) *ν*_max_ 3419, 2971, 1747, 1710, 1391, 1278, 1120, 1163, 1078, 974, 713 cm^−1^; ^1^H-NMR (CDCl_3_, 800 MHz) see Table 2; ^13^C-NMR (CDCl_3_, 200 MHz) see Table 3; (+)-LRESIMS *m*/*z* 631 [M + H]^+^, 653 [M + Na]^+^; (+)-HRESIMS *m*/*z* 653.2531 [M + Na]^+^ (calcd for C_33_H_42_O_12_Na, 653.2568).

### 3.9. Denhaminol R (**4**)

Colourless gum; ${\left[\alpha \right]}_{D}^{24}$–18.2 (*c* 0.110, MeOH); ECD *λ*_ext_ (MeOH) 216 (−5.37), 235 (0.92), 279 (−1.31) nm; UV (MeOH) *λ*_max_ (log *ε*) 224 (4.42), 281 (4.39) nm; IR (UATR) *ν*_max_ 3414, 2981, 1746, 1709, 1635, 1277, 1199, 1163, 1077, 973, 712 cm^−1^; ^1^H-NMR (CDCl_3_, 800 MHz) see Table 2; ^13^C-NMR (CDCl_3_, 200 MHz) see Table 3; (+)-LRESIMS *m*/*z* 653 [M + H]^+^; (+)-HRESIMS *m*/*z* 675.2400 [M + Na]^+^ (calcd for C_35_H_40_O_12_Na, 675.2412).

## 4. Conclusions

A UHPLC-MS method and dereplication process was developed for the rapid identification of new dihydro-β-agarofurans from Celastraceae plants. This study further exemplifies how UHPLC-MS data and database mining can be used to extract molecular features related to characteristic secondary metabolites of a plant family and thus expedite new discoveries in natural products research. This approach was successfully applied to 16 Australian Celastraceae plant extracts. Consequently, four previously undescribed dihydro-β-agarofurans (denhaminols O–R, **1**–**4**) along with a known compound denhaminol G (**5**) were successfully isolated and characterised from one prioritised plant sample, namely the fruits of *D. celastroides*. Other Celastraceae samples that were prioritised for large-scale extraction and isolation studies will be investigated in the future. The pure compounds reported in this paper will be added to the Davis Open-Access Compound Library which is housed at Compounds Australia, Griffith University [19,22,23,24,25] and will be tested in various bioassays in the future. It is worth mentioning and well-known that the chemical profile of a plant can vary due to different geographical locations and collection seasons; however, even though this research utilised only Queensland Celastraceae plants, the UHPLC-MS method developed here should be applicable to the chemical profiling of any plant sample. Furthermore, this UHPLC-MS methodology should be adaptable to the chemical investigation of any biota material, including not only plants but also microbes, marine invertebrates and fungi.

## Figures and Tables

**Table 1:** UHPLC-MS data of dihydro-β-agarofurans from *D. celastroides* fruits and scientific database analysis.

Retention Time (*t*_R_, min)	[M + H]^+^ *m*/*z*	Molecular Weight	No. of SciFinder Scholar Hits ^a^	No. of DNP Hits ^a^	Compounds ^b^
9.873	615	614	1	0	Denhaminol O (**1**)
10.097	657	656	0	0	Denhaminol P (**2**)
10.410	673	672	10	0	Denhaminol G (**5**)
11.087	631	630	4	0	Denhaminol Q (**3**)
11.513	653	652	17	0	Denhaminol R (**4**)

^a^ Accessed on the 15 June 2018. ^b^ Trivial names for new compounds identified during these studies.

**Table 2:** ^1^H (800 MHz) NMR data for denhaminols O–R (**1**–**4**) in CDCl_3_.

Position	δ_H_, multiplicity (*J* in Hz)			
	1	2	3	4
1	5.39, dd (12.1, 4.5)	5.36, dd (12.1, 4.2)	5.40, dd (12.1, 4.4)	5.46, dd (12.1, 4.4)
2*α*	1.53, m	1.51, m	1.52, m	1.54, m
2β	1.95, m	1.91, m	1.95, m	1.98, m
3*α*	1.69, ddd (13.1, 3.4, 3.4)	1.68, ddd (13.5, 3.3, 3.3)	1.68, ddd (13.0, 3.2, 3.2)	1.71, ddd (13.1, 3.4, 3.4)
3β	1.86, m	1.86, m	1.84, m	1.88, m
6	4.44, br s	5.48, s	4.37, br s	4.42, br s
7	2.37, br dd (3.5, 3.0)	2.34, br dd (3.6, 3.0)	2.64, br d (3.2)	2.73, br d (3.3)
8*α*	2.26, ddd (16.6, 7.4, 3.5)	2.46, ddd (16.6, 7.3, 3.6)	4.30, dd (6.4, 3.2)	4.35, dd (6.5, 3.3)
8β	2.03, dd (16.6, 3.0)	2.12, m	-	-
9	4.88, d (7.4)	4.92, d (7.0)	5.06, d (6.4)	5.08, d (6.5)
12	4.52, d (11.0)	4.47, d (11.1)	4.69, d (11.6)	4.88, d (11.5)
	4.69, d (11.0)	4.70, d (11.1)	4.82, d (11.6)	5.02, d (11.5)
13	1.63, s	1.58, s	1.65, s	1.76, s
14	1.59, s	1.33, s	1.57, s	1.60, s
15	1.33, s	1.36, s	1.32, s	1.35, s
17	4.31, d (15.9)	4.33 (15.8)	4.33, d (15.8)	4.34, d (15.9)
	4.45, d (15.9)	4.46 (15.8)	4.47, d (15.8)	4.47, d (15.9)
19	1.88, s	1.91, s	1.92, s	1.91, s
21	6.40, d (16.0)	6.39, d (16.0)	6.49, d (15.9)	6.54, d (16.0)
22	7.65, d (16.0)	7.66, d (16.0)	7.69, d (15.9)	7.71, d (16.0)
24	7.56, m	7.56, m	7.58, m	7.54, m
25	7.36, m	7.37, m	7.36, m	7.33, m
26	7.36, m	7.37, m	7.36, m	7.36, m
27	7.36, m	7.37, m	7.36, m	7.33, m
28	7.56, m	7.56, m	7.58, m	7.54, m
31	6.81, m	6.81, m	6.85, m	8.03, m
32	1.78, m	1.78, m	1.80, m	7.45, m
33	1.79, m	1.78, m	1.82, m	7.57, m
34	-	-	-	7.45, m
35	-	2.15, s	-	8.03, m

**Table 3:** ^13^C (200 MHz) NMR data for denhaminols O–R (**1**–**4**) in CDCl_3._

Position	δ_H_, Type			
	1	2	3	4
**1**	73.2,* CH	73.5, CH	73.2,* CH	73.2,* CH
**2**	23.5, CH_2_	23.6, CH_2_	23.3, CH_2_	23.3, CH_2_
**3**	36.9, CH_2_	38.3, CH_2_	37.0, CH_2_	37.0, CH_2_
**4**	73.3,* C	70.6, C	73.3,* CH	73.3,* CH
**5**	92.4, C	92.4, C	91.8, C	91.9, C
**6**	79.4, CH	79.4, CH	78.0, CH	78.0, CH
**7**	49.0, CH	48.0, CH	56.0, CH	55.9, CH
**8**	31.7, CH_2_	31.8, CH_2_	70.3, CH	70.5, CH
**9**	72.1, CH	72.0, CH	74.4, CH	74.6, CH
**10**	50.3, C	51.6, C	48.7, C	48.8, C
**11**	84.9, C	84.8, C	85.5, C	85.6, C
**12**	69.4, CH_2_	68.8, CH_2_	69.8, CH_2_	70.3, CH_2_
**13**	25.1, CH_3_	24.7, CH_3_	24.9, CH_3_	24.9, CH_3_
**14**	23.8, CH_3_	24.2, CH_3_	23.8, CH_3_	23.8, CH_3_
**15**	20.0, CH_3_	19.8, CH_3_	20.1, CH_3_	20.2, CH_3_
**16**	167.1, C	167.1, C	167.1, C	167.1, C
**17**	60.8, CH_2_	60.8, CH_2_	60.8, CH_2_	60.8, CH_2_
**18**	170.2, C	170.2, C	170.2, C	170.2, C
**19**	20.3, CH_3_	20.4, CH_3_	20.3, CH_3_	20.3, CH_3_
**20**	165.9, C	166.0, C	167.6, C	167.9, C
**21**	117.7, CH	117.0, CH	117.5, CH	117.3, CH
**22**	146.2, CH	146.3, CH	146.9, CH	147.1, CH
**23**	134.6, C	134.6, C	134.6, C	134.5, C
**24**	128.6, CH	128.7, CH	128.7, CH	128.76, CH
**25**	128.8, CH	128.8, CH	128.9, CH	128.83, CH
**26**	130.4, CH	130.5, CH	130.5, CH	130.6, CH
**27**	128.8, CH	128.8, CH	128.9, CH	128.83, CH
**28**	128.6, CH	128.7, CH	128.7, CH	128.76, CH
**29**	167.6, C	127.5, C	167.9, C	166.4, C
**30**	128.5, C	128.4, C	128.8, C	130.5, C
**31**	137.7, CH	137.9, CH	137.5, CH	129.8, CH
**32**	14.5, CH_3_	14.6, CH_3_	14.5, CH_3_	128.5, CH
**33**	12.2, CH_3_	12.2, CH_3_	12.2, CH_3_	133.1, CH
**34**	-	170.6, C	-	128.5, CH
**35**	-	21.8, CH_3_	-	129.8, CH

* Interchangeable signals.

## Supplementary Materials

The following materials are available online at https://www.mdpi.com/1420-3049/24/5/859/s1: 1D/2D NMR spectra of compounds **1**–**4**, ECD spectra of **1**–**5** and diagnostic 2D correlations for compounds **2**–**4**; Australian Celastraceae plant collection date, location and voucher specimen codes.
