# Supplementary material for: Using UHPLC-MS Profiling for the Discovery of New Dihydro-β-Agarofurans from Australian Celastraceae Plant Extracts
Source: Molecules. 2019 Feb 28;24(5):859. doi: 10.3390/molecules24050859 (PMC6429220; doi:10.3390/molecules24050859)
Supplement: Supplementary file 1 [file molecules-24-00859-s001.pdf]

Supplementary data contents page

# Using UHPLC-MS profiling for the discovery of new dihydro-β-agarofurans from Australian Celastraceae plant extracts

**Mario Wibowo [1], Paul I. Forster [2], Gordon P. Guymer [2], Andreas Hofmann [1, 3], and Rohan A. Davis [1, *]**

[1] Griffith Institute for Drug Discovery, Griffith University, Brisbane, QLD 4111, Australia
[2] Queensland Herbarium, Brisbane Botanic Gardens, Toowong, QLD 4066, Australia
[3] Department of Veterinary Biosciences, Melbourne Veterinary School, The University of Melbourne, Melbourne, VIC 3010, Australia

**Corresponding Author Contact Details:**
Tel.: +61 7 3735 6043. Fax: +61 7 3735 6001. E-mail: r.davis@griffith.edu.au

**S1**. UHPLC chromatograms of 16 $CH_2Cl_2$ extracts of Australian Celastraceae plants
**S2**. UHPLC-MS data of the major UV peaks of 16 $CH_2Cl_2$ extracts of Australian Celastraceae and scientific database analyses
**S3**. $^1$H NMR (800 MHz) spectrum of denhaminol O (**1**) in $CDCl_3$
**S4**. $^{13}$C NMR (200 MHz) spectrum of denhaminol O (**1**) in $CDCl_3$
**S5**. COSY spectrum of denhaminol O (**1**) in $CDCl_3$
**S6**. HSQC spectrum of denhaminol O (**1**) in $CDCl_3$
**S7**. HMBC spectrum of denhaminol O (**1**) in $CDCl_3$
**S8**. ROESYspectrum of denhaminol O (**1**) in $CDCl_3$
**S9**. $^1$H NMR (800 MHz) spectrum of denhaminol P (**2**) in $CDCl_3$
**S10**. $^{13}$C NMR (200 MHz) spectrum of denhaminol P (**2**) in $CDCl_3$
**S11**. COSY spectrum of denhaminol P (**2**) in $CDCl_3$
**S12**. HSQC spectrum of denhaminol P (**2**) in $CDCl_3$
**S13**. HMBC spectrum of denhaminol P (**2**) in $CDCl_3$
**S14**. ROESYspectrum of denhaminol P (**2**) in $CDCl_3$
**S15**. $^1$H NMR (800 MHz) spectrum of denhaminol Q (**3**) in $CDCl_3$
**S16**. $^{13}$C NMR (200 MHz) spectrum of denhaminol Q (**3**) in $CDCl_3$
**S17**. COSY spectrum of denhaminol Q (**3**) in $CDCl_3$
**S18**. HSQC spectrum of denhaminol Q (**3**) in $CDCl_3$
**S19**. HMBC spectrum of denhaminol Q (**3**) in $CDCl_3$
**S20**. ROESYspectrum of denhaminol Q (**3**) in $CDCl_3$
**S21**. $^1$H NMR (800 MHz) spectrum of denhaminol R (**4**) in $CDCl_3$
**S22**. $^{13}$C NMR (200 MHz) spectrum of denhaminol R (**4**) in $CDCl_3$
**S23**. COSY spectrum of denhaminol R (**4**) in $CDCl_3$
**S24**. HSQC spectrum of denhaminol R (**4**) in $CDCl_3$
**S25**. HMBC spectrum of denhaminol R (**4**) in $CDCl_3$
**S26**. ROESYspectrum of denhaminol R (**4**) in $CDCl_3$

**S27**. ECD spectra of denhaminols O–R (**1**–**4**) and denhaminol G (**5**) in MeOH
**S28**. Diagnostic 2D NMR correlations for denhaminol P (**2**)
**S29**. Diagnostic 2D NMR correlations for denhaminol Q (**3**)
**S30**. Diagnostic 2D NMR correlations for denhaminol R (**4**)
**S31**. Australian Celastraceae plant collection date, location, and voucher specimen codes

**S1** UHPLC chromatograms of 16 $CH_2Cl_2$ extracts of Australian Celastraceae plants

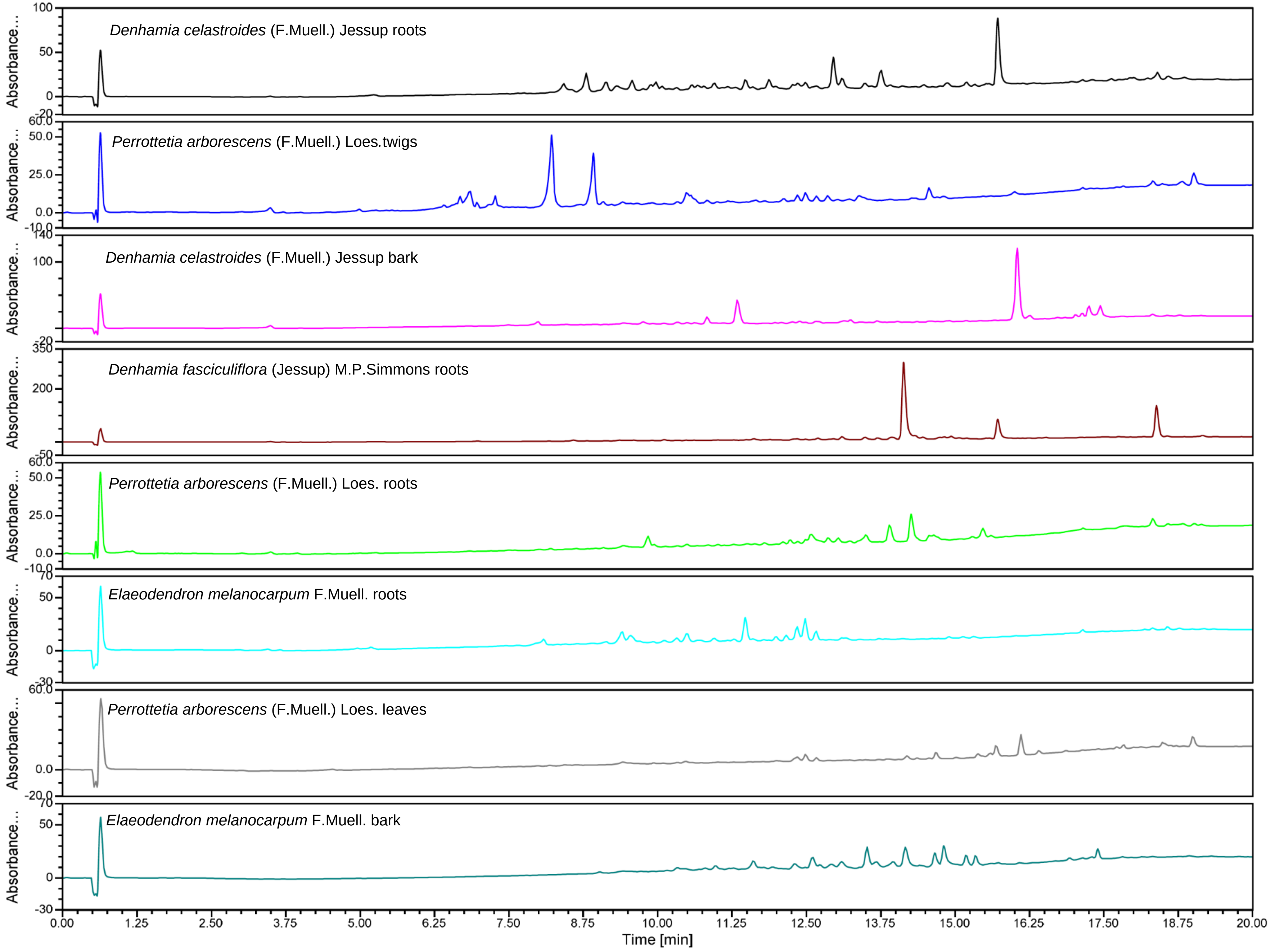

**S1 Cont'd** UHPLC chromatograms of 16 $CH_2Cl_2$ extracts of Australian Celastraceae plants

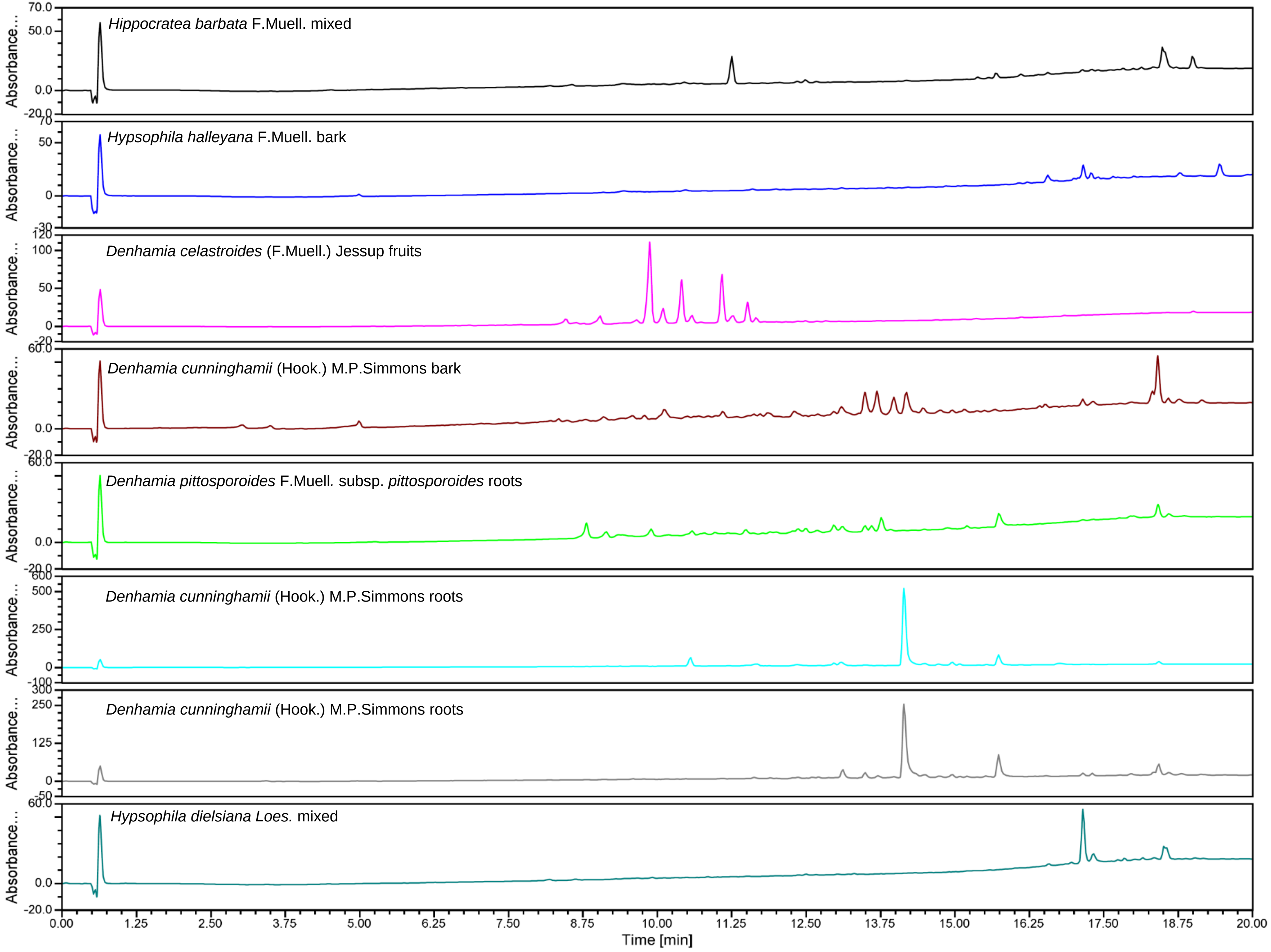

**S2** UHPLC-MS data of the major UV peaks of 16 $CH_2Cl_2$ extracts of Australian Celastraceae and scientific database analyses

| Scientific name | Sample type | Retention time ($t_R$, min) | $[M+H]^+$ *m/z* | $[M–H]^-$ *m/z* | Molecular weight | No. of SciFinder Scholar hits[a] | No. of DNP hits[a] |
|---|---|---|---|---|---|---|---|
| *Denhamia celastroides* (F.Muell.) Jessup | Roots | 12.96 | 497 | – | 496 | 6 | 0 |
| | | 13.11 | 481 | – | 480 | 8 | 0 |
| | | 13.77 | 481 | – | 480 | 8 | 0 |
| | | 15.72 | 465 | – | 464 | 17 | 0 |
| *Perrottetia arborescens* (F.Muell.) Loes | Twigs | 8.20 | 457 | – | 456 | 29 | 0 |
| | | 8.92 | 441 | – | 440 | 21 | 0 |
| *Denhamia celastroides* (F.Muell.) Jessup | Bark | 11.32 | – | 315 | 316 | 6 | 0 |
| | | 16.04 | – | 603 | 604 | 4 | 0 |
| *Denhamia fasciculiflora* (Jessup) M.P.Simmons | Roots | 14.15 | 451 | – | 450 | 9 | 0 |
| | | 15.72 | 465 | – | 464 | 17 | 0 |
| | | 18.40 | 303 | – | 302 | 13 | 0 |
| *Perrottetia arborescens* (F.Muell.) Loes. | Roots | 13.49 | – | 569 | 570 | 3 | 0 |
| | | 13.92 | – | 617 | 618 | 1 | 0 |

| | | | | | | | |
|---|---|---|---|---|---|---|---|
| | | 14.26 | – | 615 | 616 | 3 | 0 |
| | | 15.45 | – | 573 | 573 | 0 | 0 |
| *Elaeodendron melanocarpum* F.Muell. | Roots | 11.49 | – | 471 | 472 | 28 | 0 |
| | | 12.32 | – | – | – | – | – |
| | | 12.94 | – | – | – | – | – |
| | | 12.66 | – | – | – | – | – |
| *Perrottetia arborescens* (F.Muell.) Loes. | Leaves | – | – | – | – | – | – |
| | | – | – | – | – | – | – |
| | | 16.10 | 593 | – | 592 | 9 | 0 |
| *Elaeodendron melanocarpum* F.Muell. | Bark | 14.17 | – | 621 | 622 | 4 | 0 |
| | | 14.64 | – | 471 | 472 | 28 | 0 |
| *Hippocratea barbata* F.Muell. | Mixed | 11.24 | 507 | – | 506 | 8 | 0 |
| | | 18.48 | – | – | – | – | – |
| | | 18.99 | – | – | – | – | – |
| *Hypsophila halleyana* F.Muell. | Bark | 17.17 | 615 | – | 614 | 1 | 0 |
| | | 17.29 | 629 | – | 628 | 0 | 0 |
| *Denhamia* | Fruits | 9.87 | 615 | – | 614 | 1 | 0 |

| | | | | | | | |
|---|---|---|---|---|---|---|---|
| *celastroides* (F.Muell.) Jessup | | | | | | | |
| | | 10.09 | 657 | – | 656 | 0 | 0 |
| | | 10.41 | 673 | – | 672 | 10 | 0 |
| | | 11.03 | 631 | – | 630 | 4 | 0 |
| | | 11.51 | 653 | – | 652 | 17 | 0 |
| *Denhamia cunninghamii* (Hook.) M.P.Simmons | Bark | 13.70 | – | 447 | 448 | 7 | 0 |
| | | 13.96 | – | 547 | 548 | 11 | 0 |
| | | 14.17 | – | 469 | 470 | 14 | 0 |
| | | 18.4 | – | 383 | 384 | 6 | 0 |
| *Denhamia pittosporoides* F.Muell. subsp. *pittosporoides* | Roots | 8.82 | – | 361 | 362 | 5 | 0 |
| | | 13.75 | – | 497 | 498 | 10 | 0 |
| | | 15.72 | 465 | – | 464 | 17 | 0 |
| *Denhamia cunninghamii* (Hook.) M.P.Simmons | Roots | 10.54 | 319 | – | 318 | 7 | 0 |
| | | 14.15 | 451 | – | 450 | 9 | 0 |
| | | 15.72 | 465 | – | 464 | 17 | 0 |

| | | | | | | | |
|---|---|---|---|---|---|---|---|
| *Denhamia cunninghamii* (Hook.) M.P.Simmons | Roots | 14.15 | 451 | – | 450 | 9 | 0 |
| | | 15.72 | 465 | – | 464 | 17 | 0 |
| | | 18.40 | 429 | – | 428 | 12 | 0 |
| *Hypsophila dielsiana* Loes. | Mixed | 17.17 | 441 | – | 440 | 21 | 0 |

**S3** $^{1}$H NMR (800 MHz) spectrum of denhaminol O (**1**) in $CDCl_3$

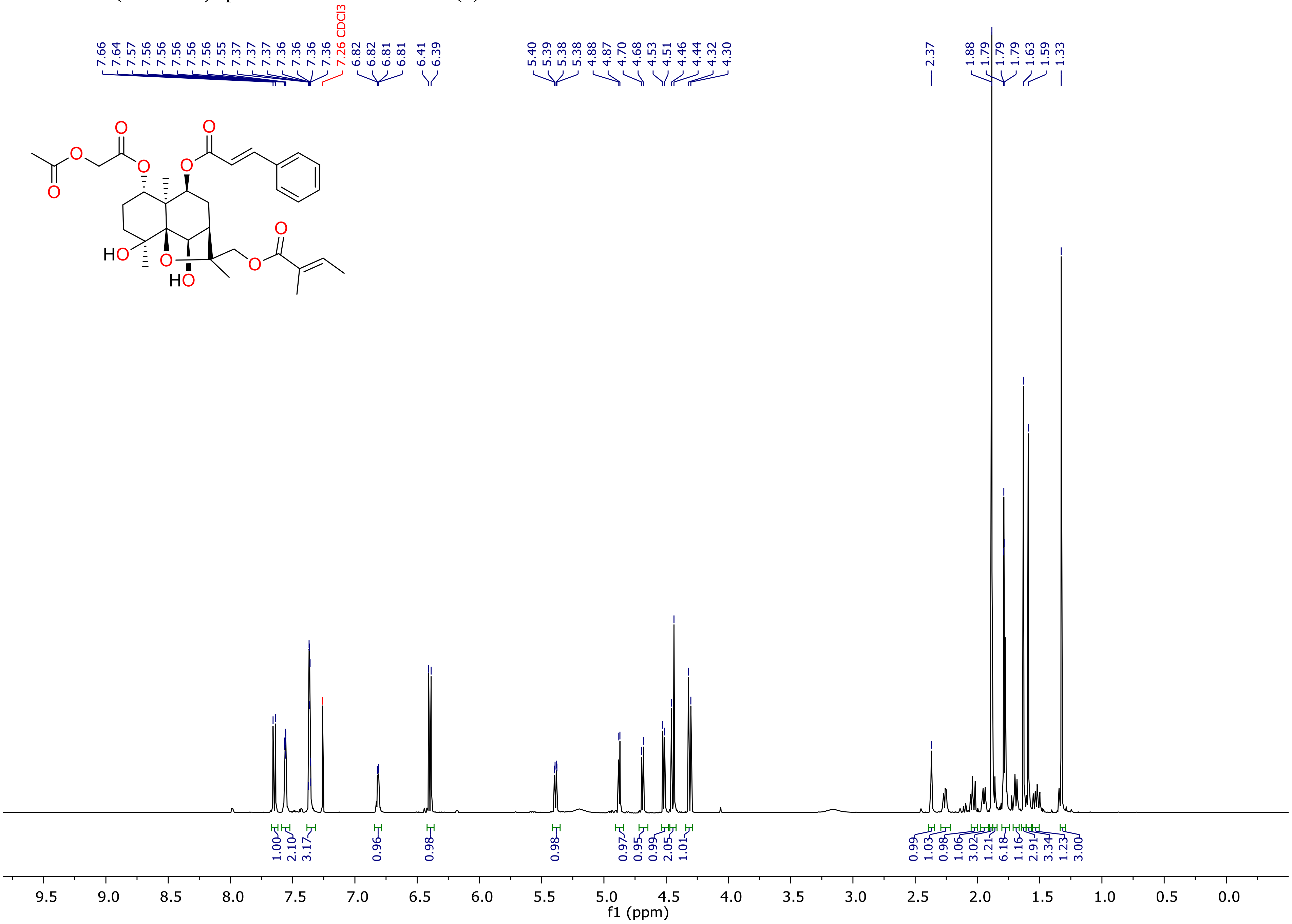

**S4** $^{13}$C NMR (200 MHz) spectrum of denhaminol O (**1**) in $CDCl_3$

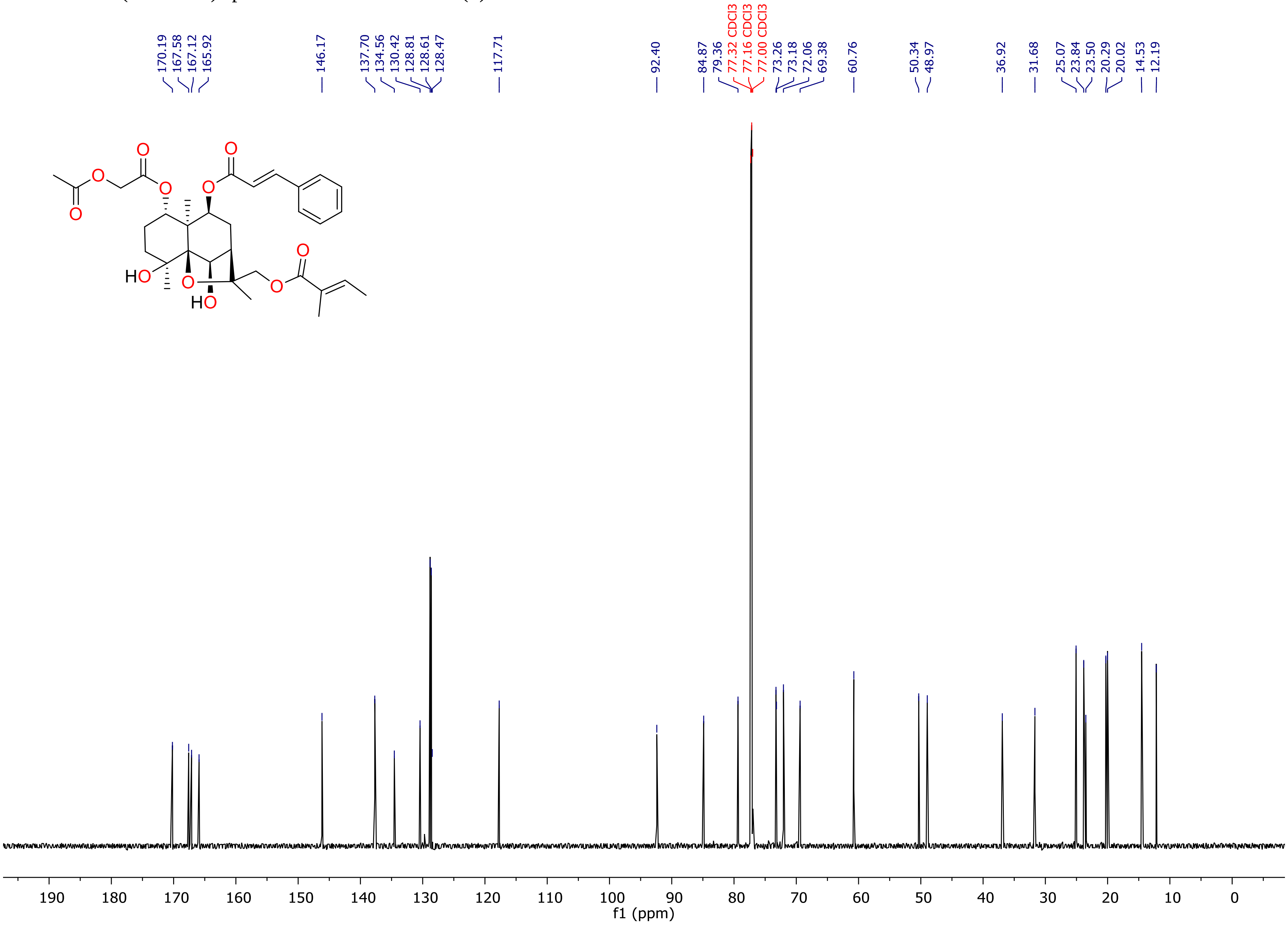

**S5** COSY spectrum of denhaminol O (**1**) in $CDCl_3$

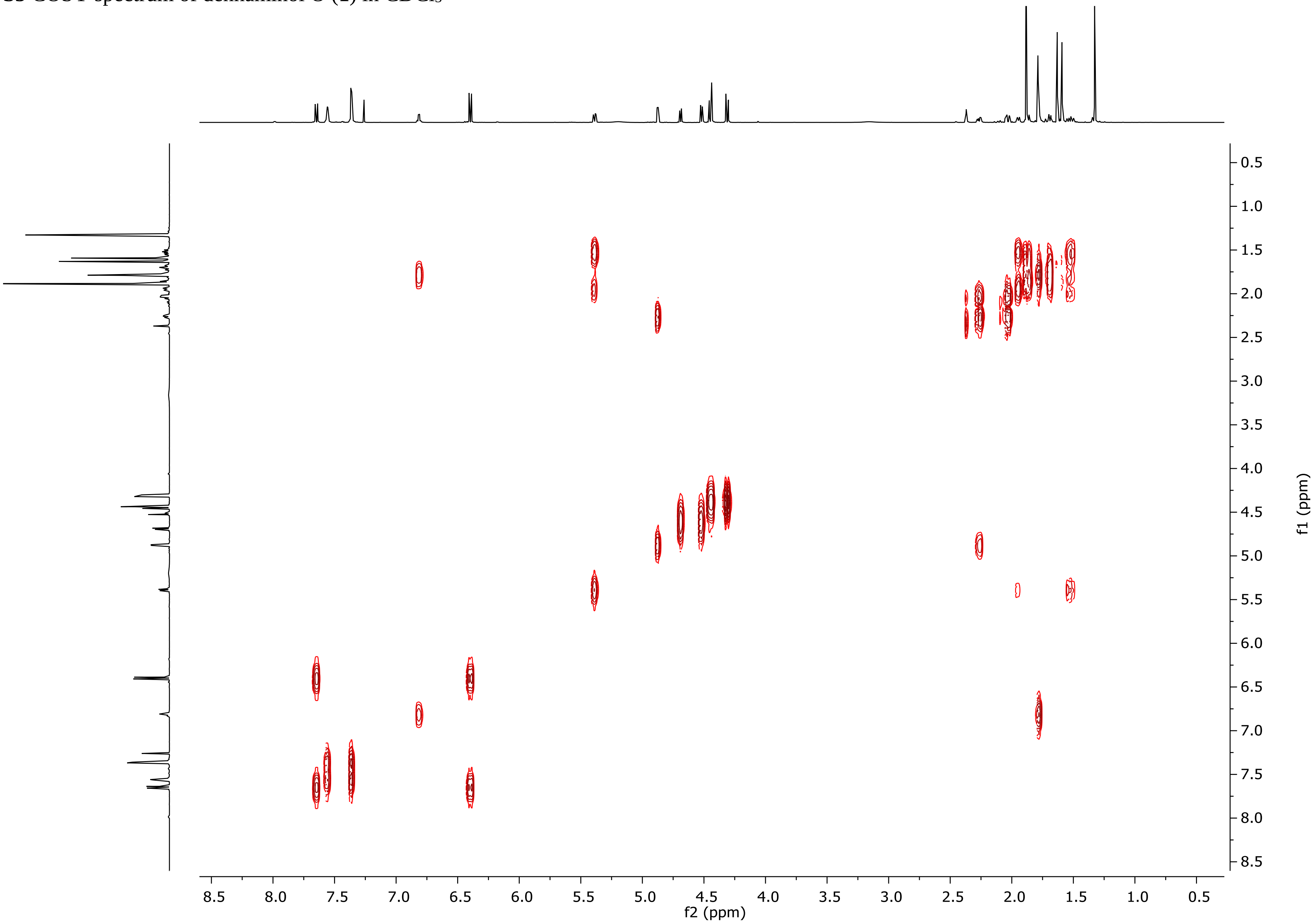

**S6** HSQC spectrum of denhaminol O (**1**) in $CDCl_3$

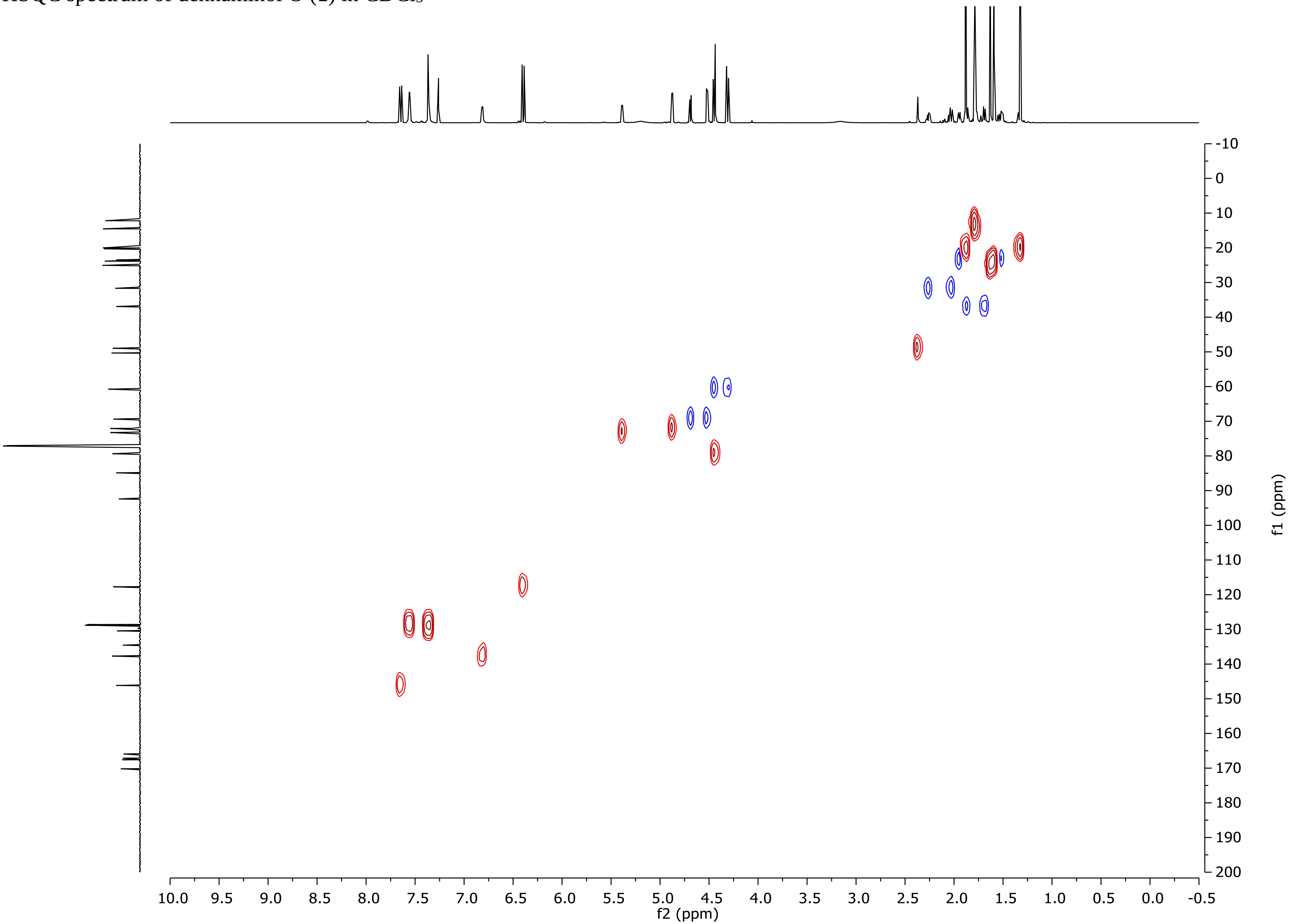

**S7** HMBC spectrum of denhaminol O (**1**) in $CDCl_3$

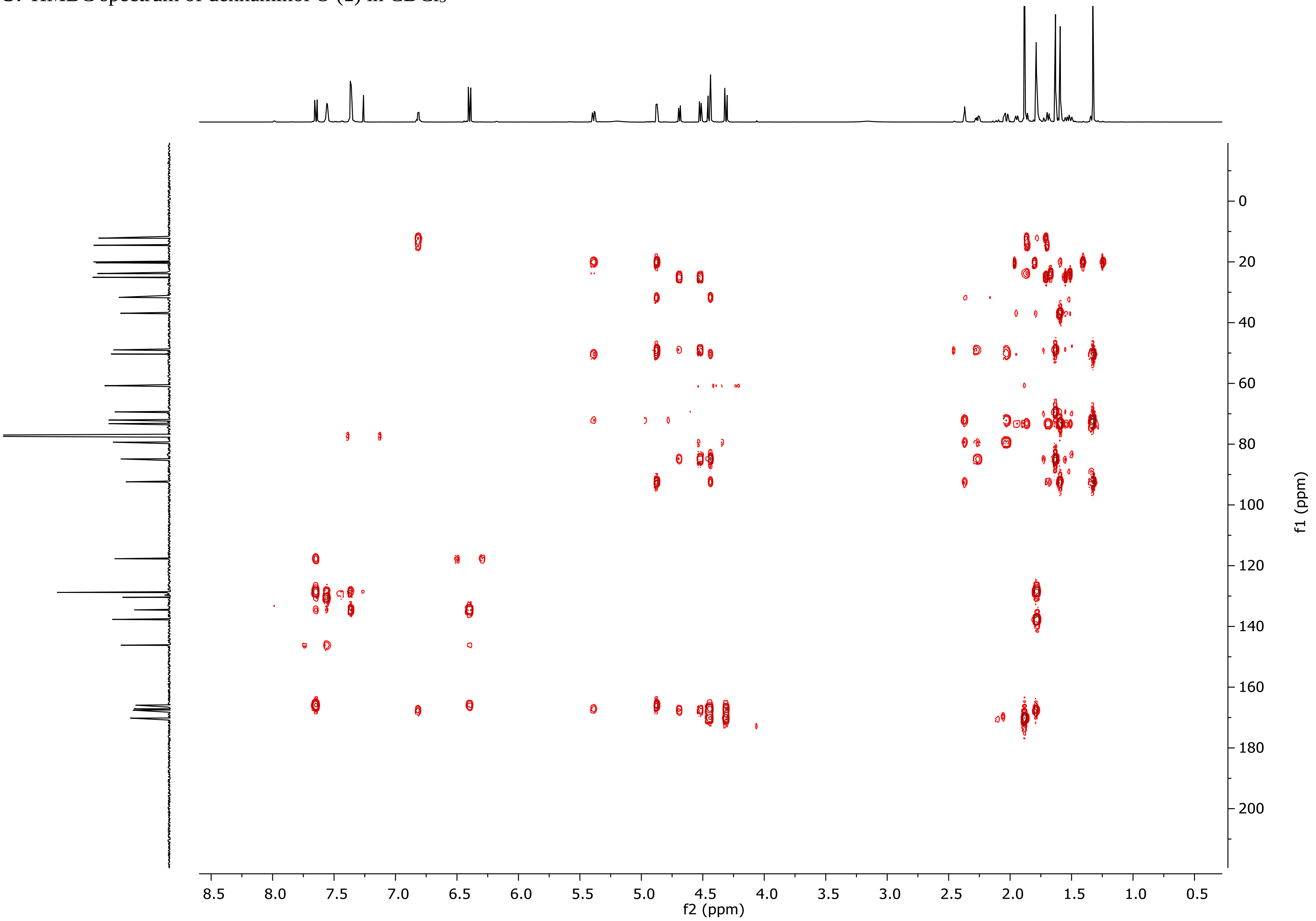

**S8** ROESY spectrum of denhaminol O (**1**) in $CDCl_3$

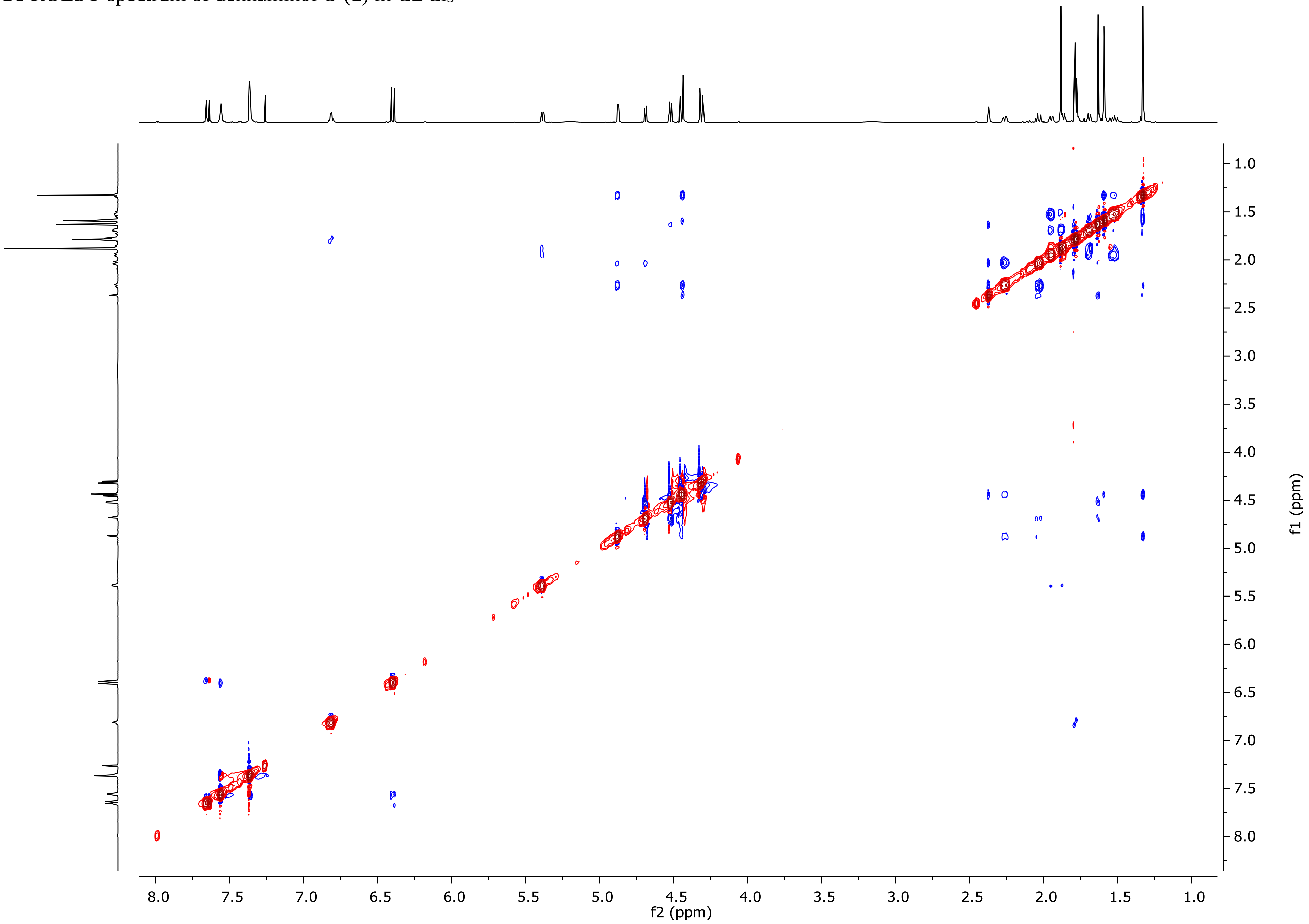

**S9** $^{1}H$ NMR (800 MHz) spectrum of denhaminol P (**2**) in $CDCl_3$

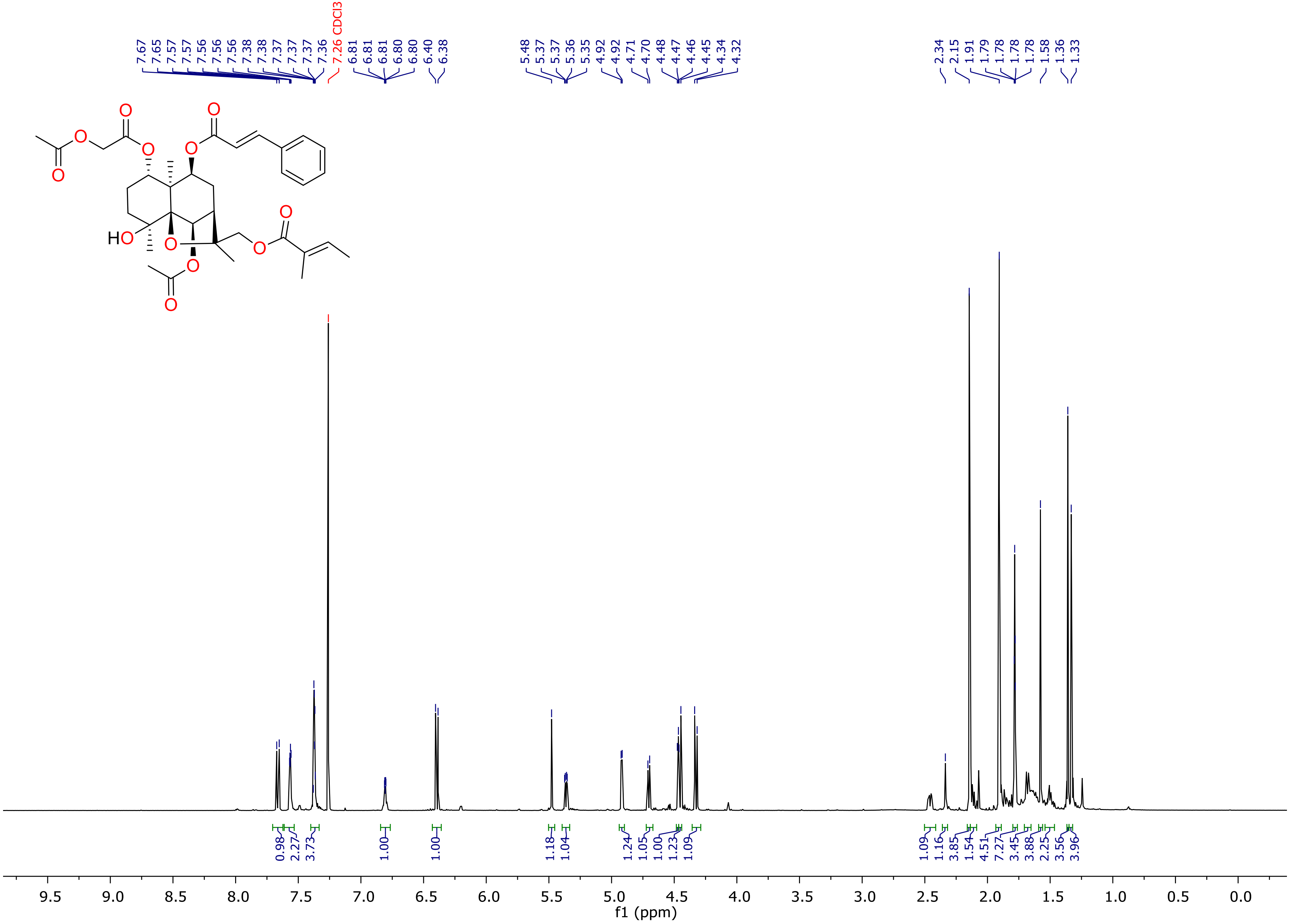

**S10** $^{13}$C NMR (200 MHz) spectrum of denhaminol P (**2**) in $CDCl_3$

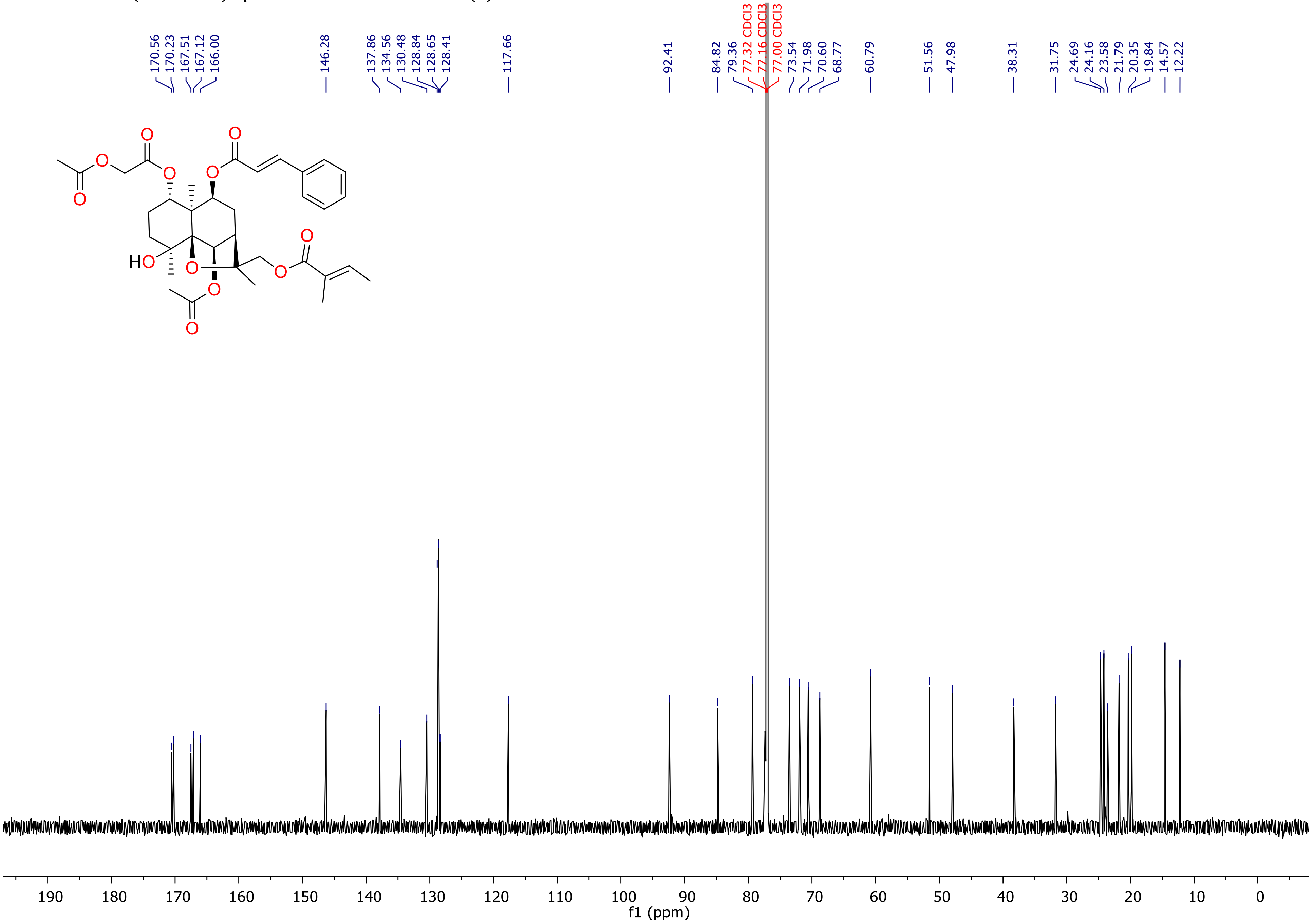

**S11** COSY spectrum of denhaminol P (**2**) in $CDCl_3$

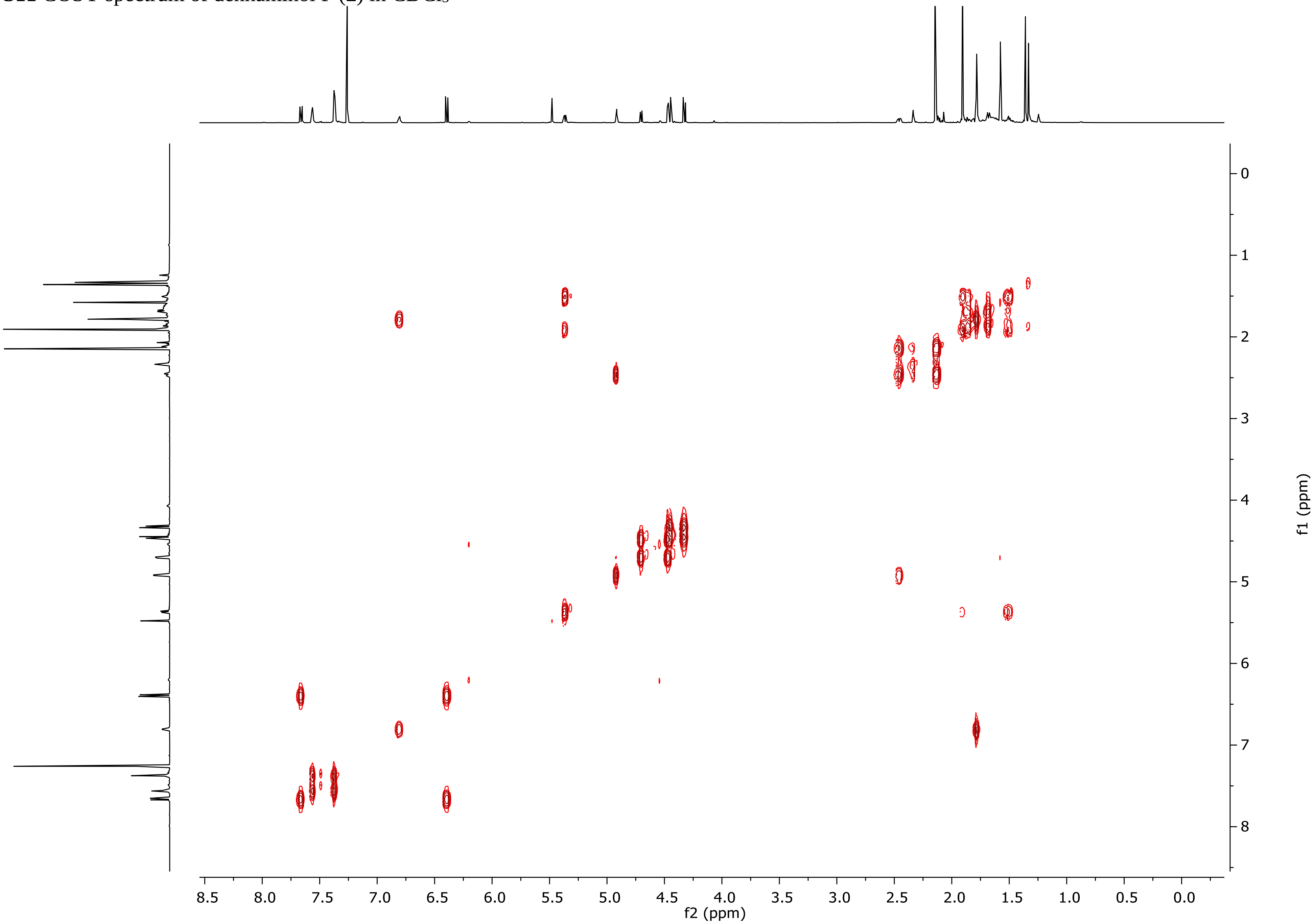

**S12** HSQC spectrum of denhaminol P (**2**) in $CDCl_3$

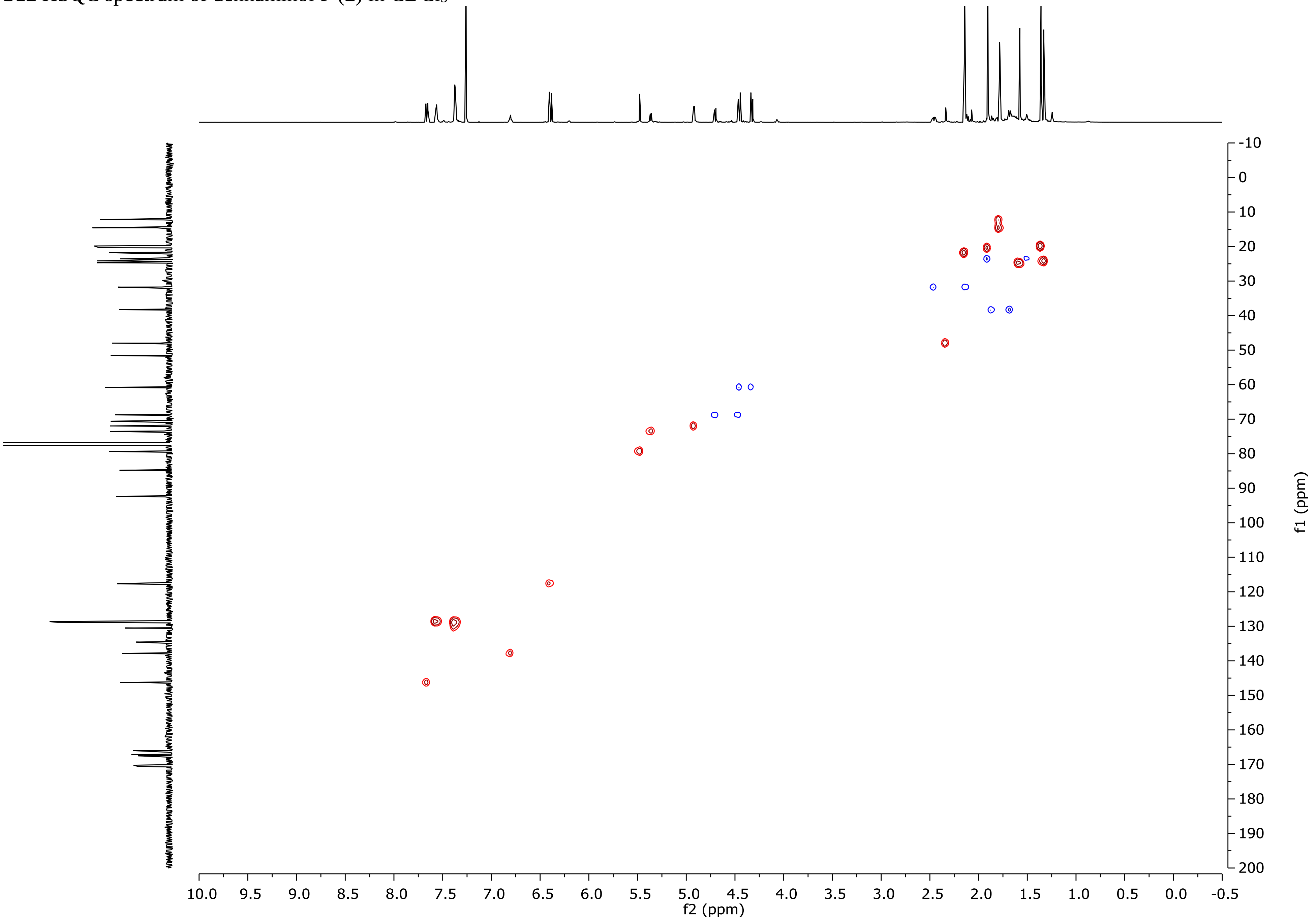

**S13** HMBC spectrum of denhaminol P (**2**) in $CDCl_3$

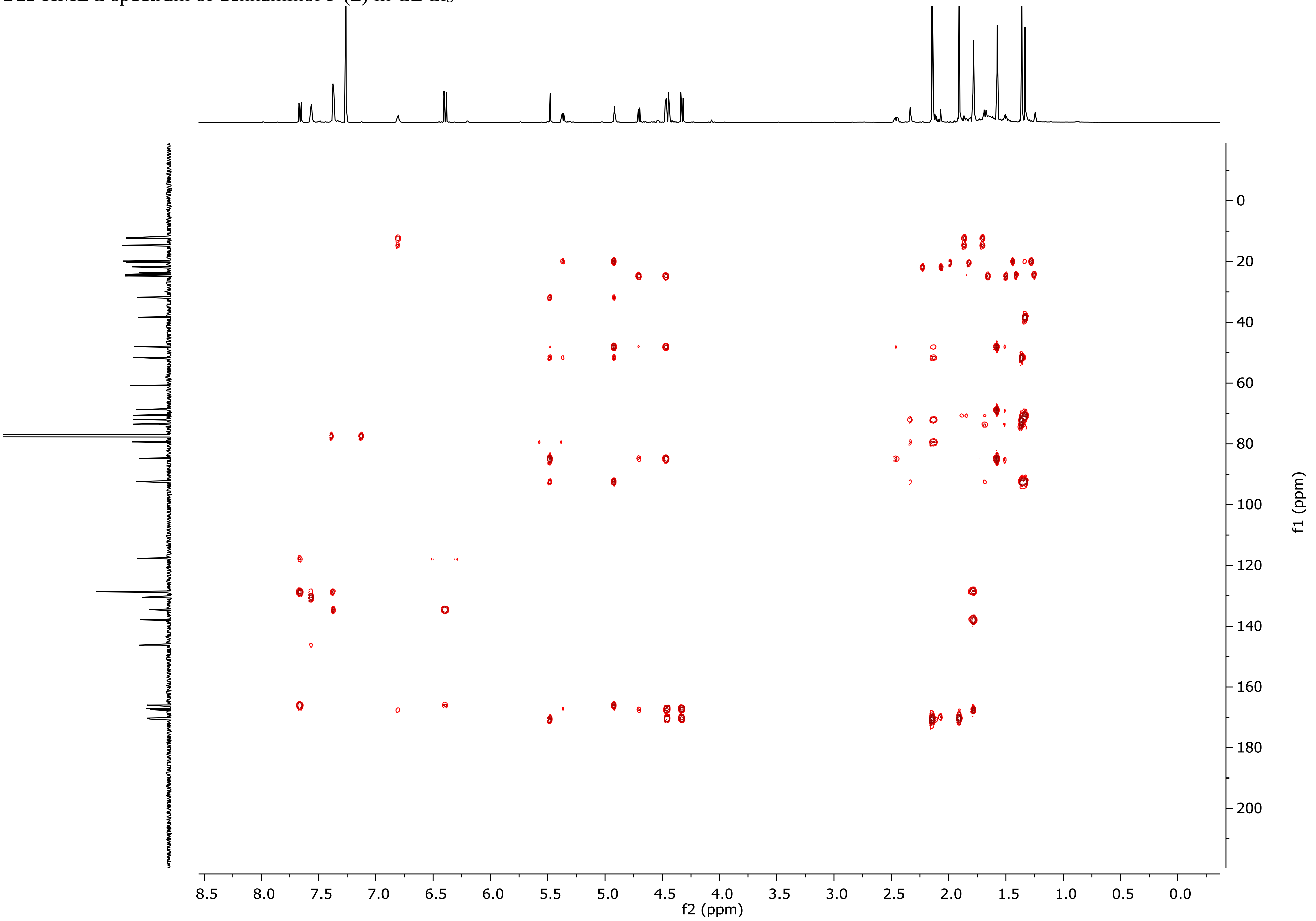

**S14** ROESY spectrum of denhaminol P (**2**) in $CDCl_3$

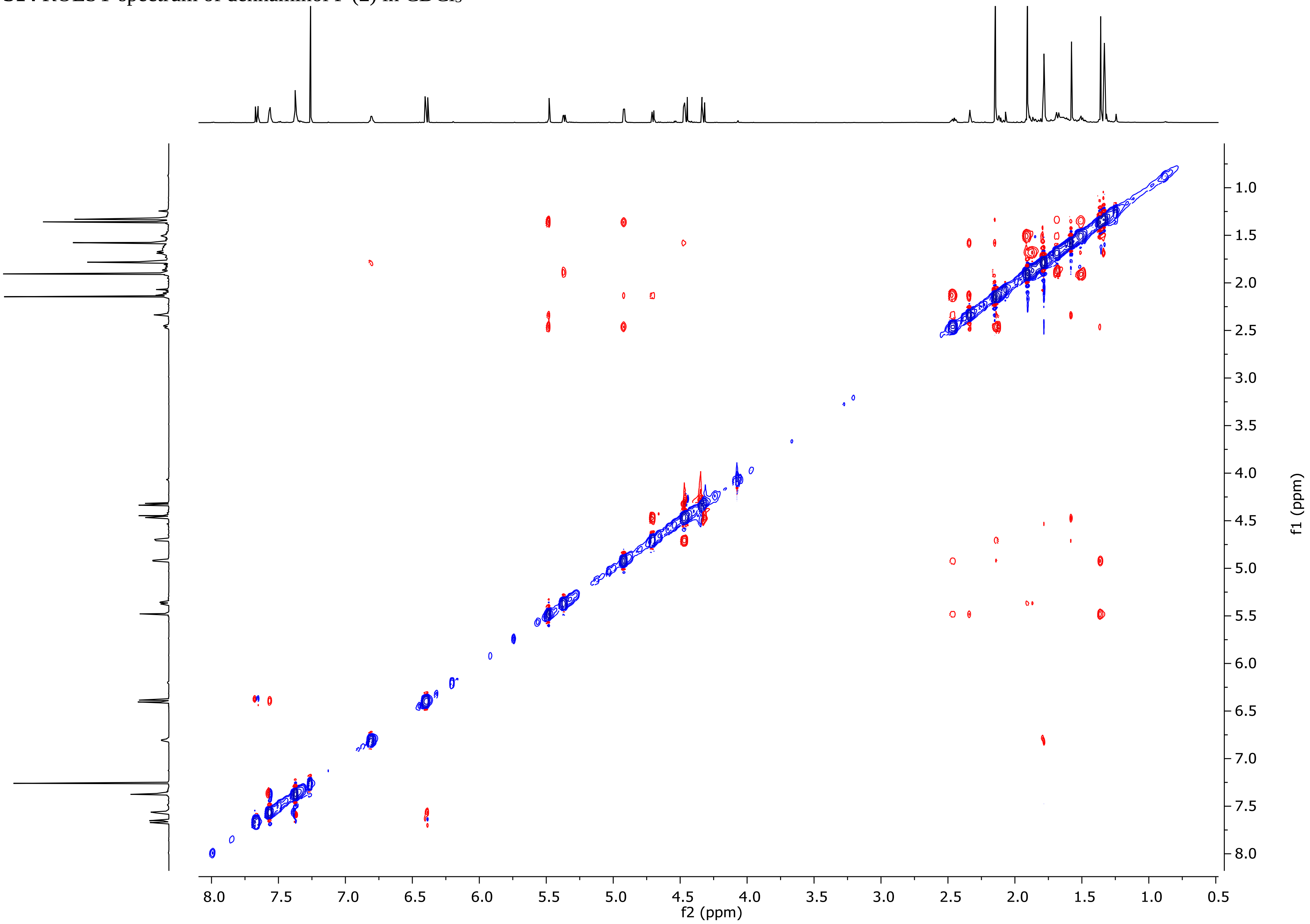

**S15** $^{1}$H NMR (800 MHz) spectrum of denhaminol Q (**3**) in $CDCl_3$

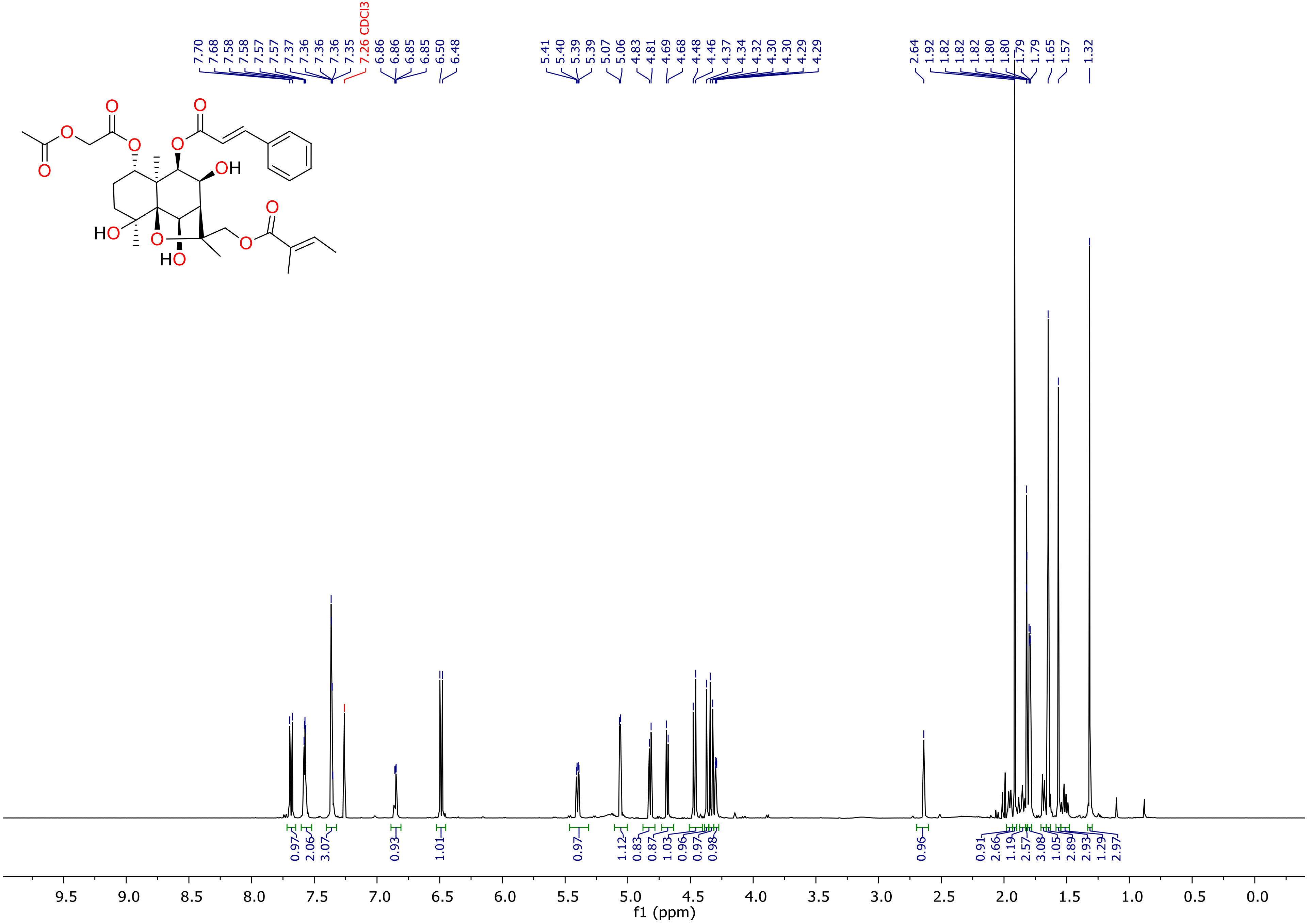

**S16** $^{13}$C NMR (200 MHz) spectrum of denhaminol Q (**3**) in $CDCl_3$

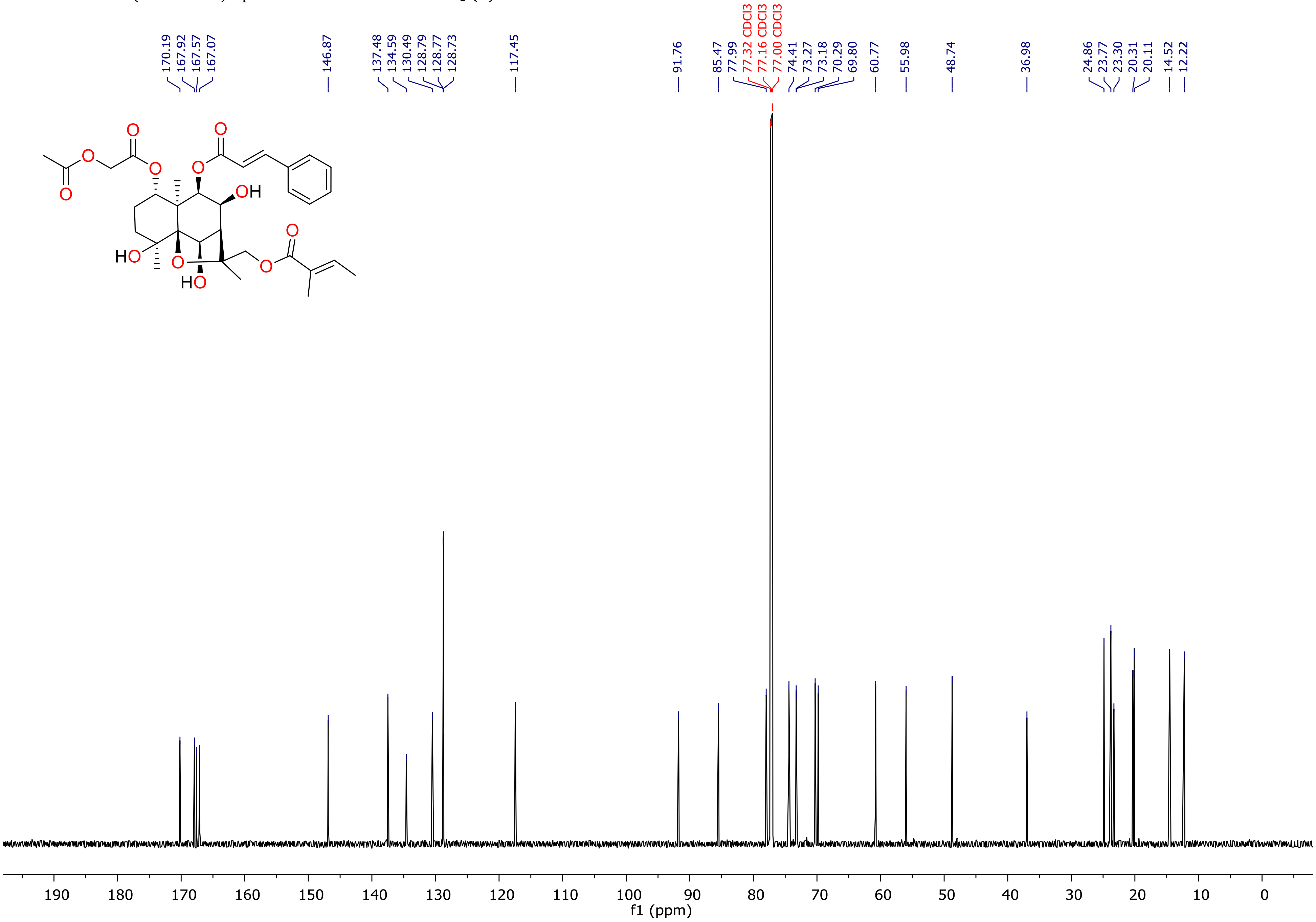

**S17** COSY spectrum of denhaminol Q (**3**) in $CDCl_3$

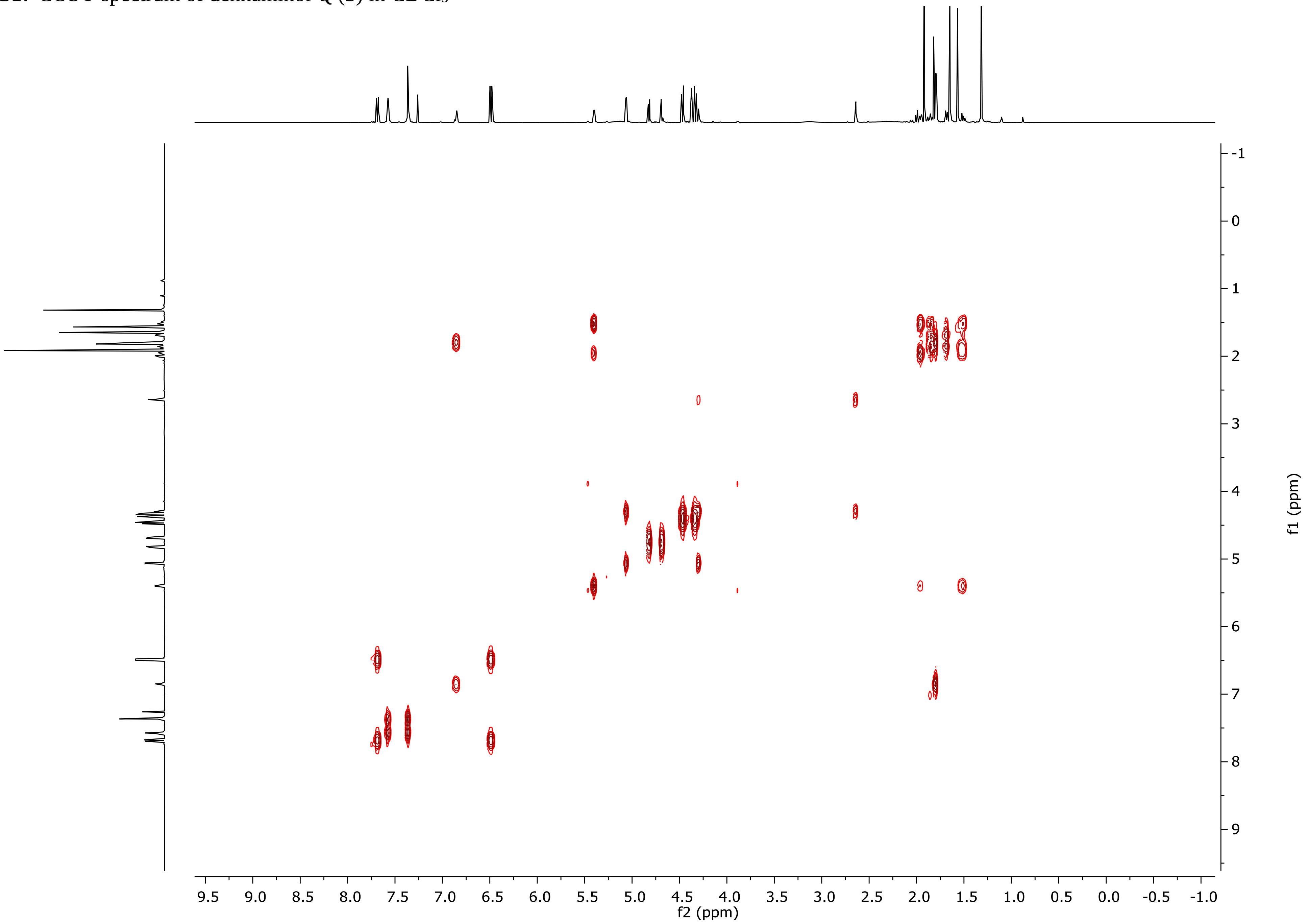

**S18** HSQC spectrum of denhaminol Q (**3**) in $CDCl_3$

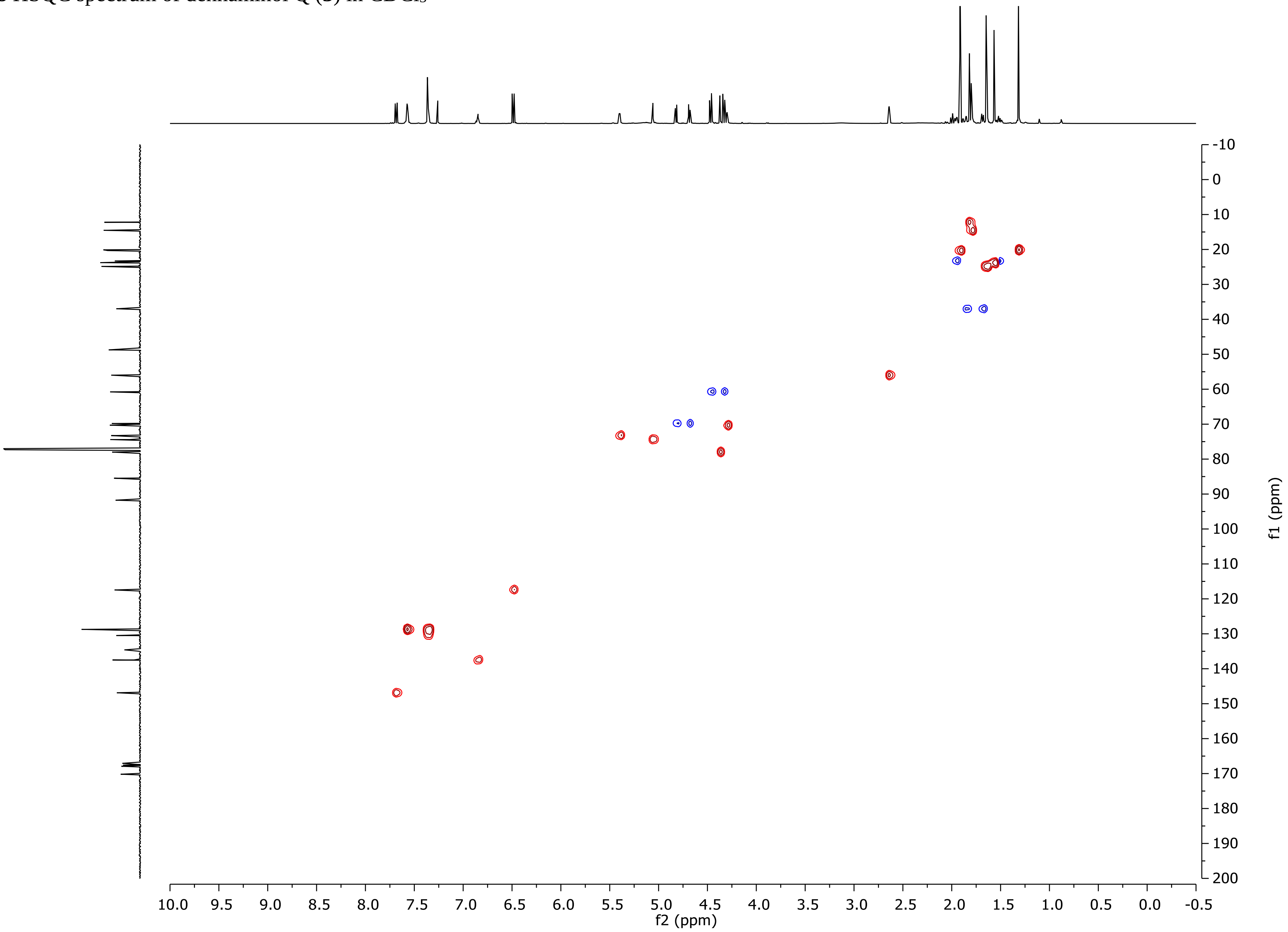

**S19** HMBC spectrum of denhaminol Q (**3**) in $CDCl_3$

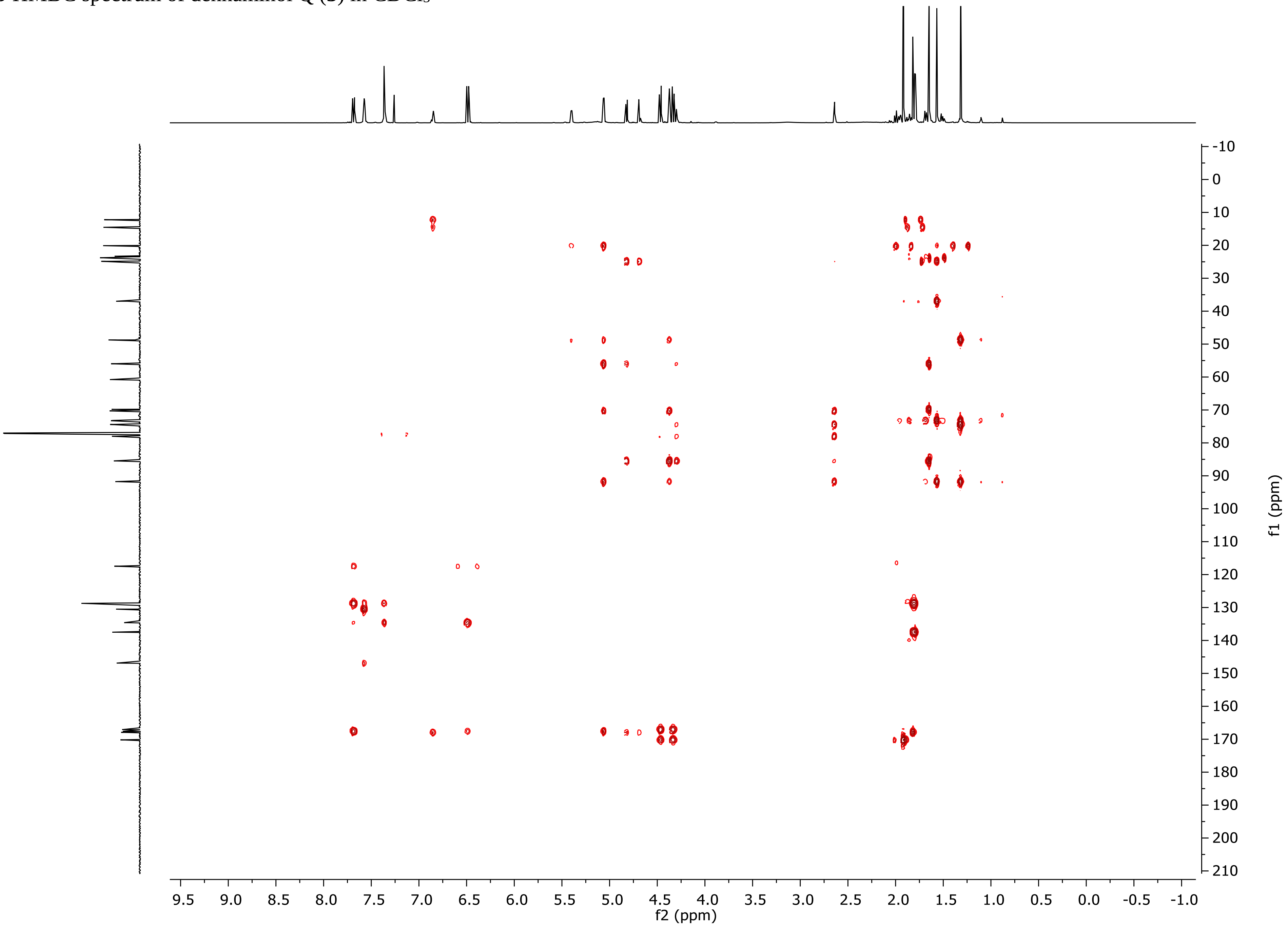

**S20** ROESY spectrum of denhaminol Q (**3**) in $CDCl_3$

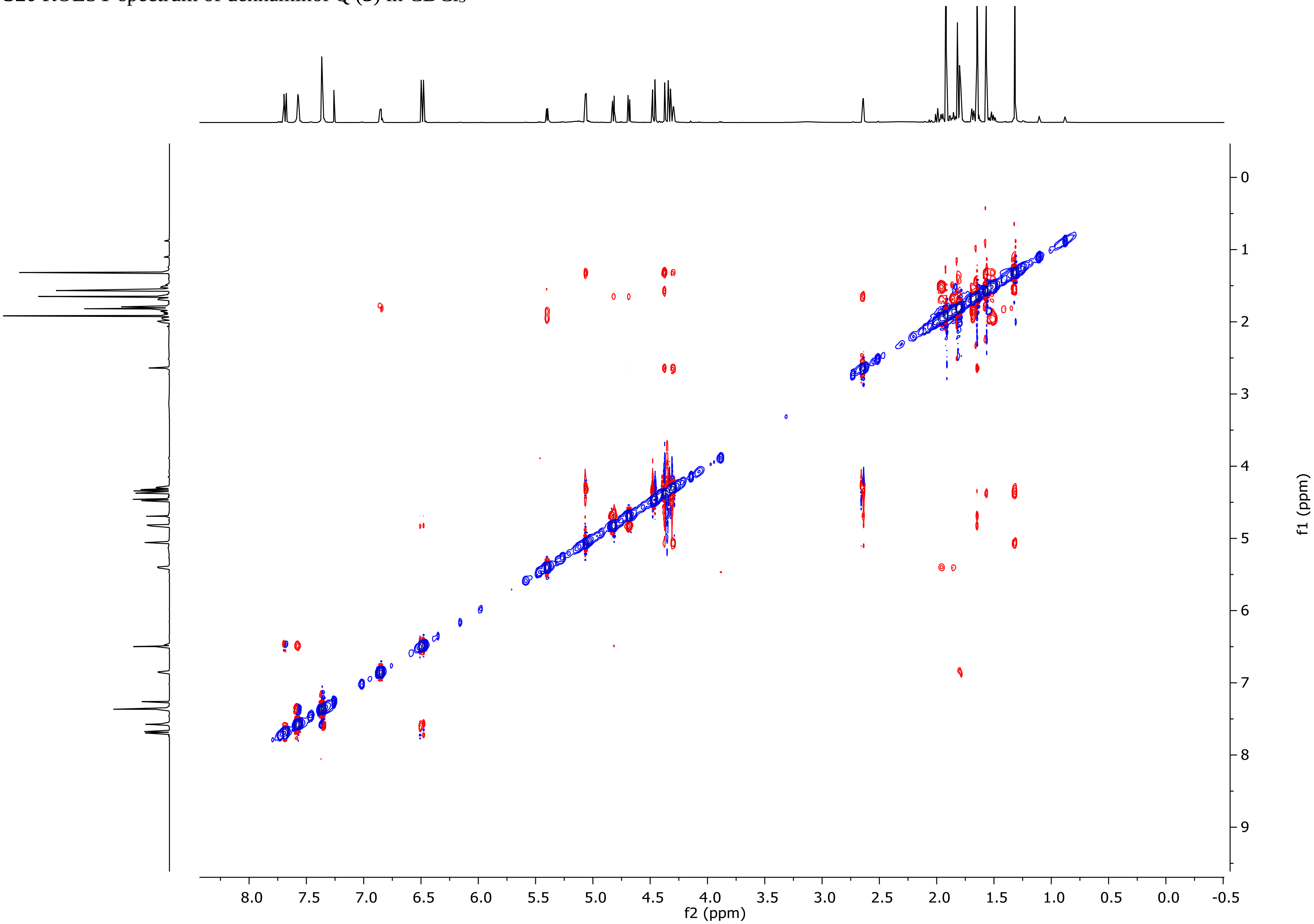

**S21** $^{1}$H NMR (800 MHz) spectrum of denhaminol R (**4**) in $CDCl_3$

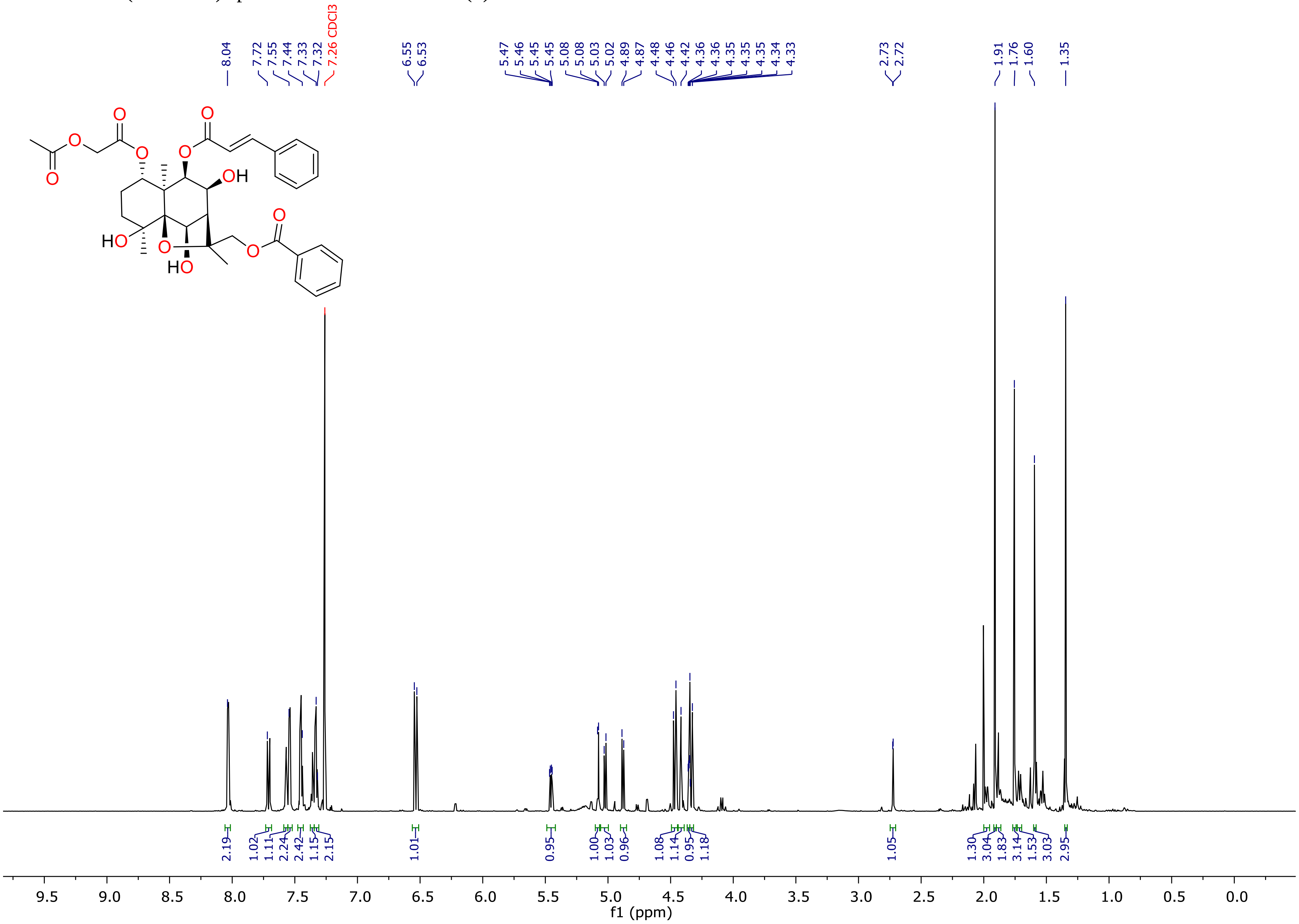

**S22** $^{13}$C NMR (200 MHz) spectrum of denhaminol R (**4**) in $CDCl_3$

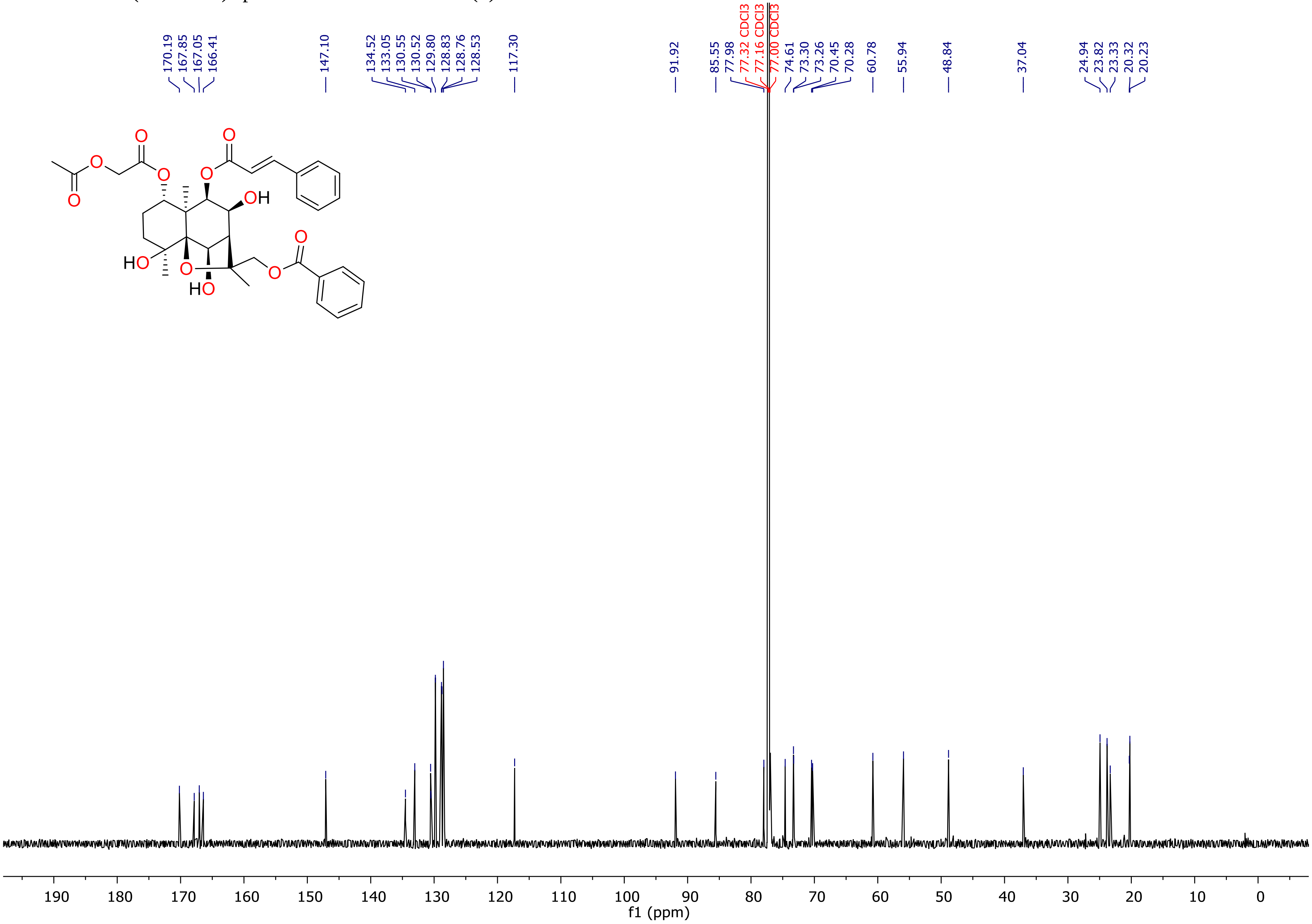

**S23** COSY spectrum of denhaminol R (**4**) in $CDCl_3$

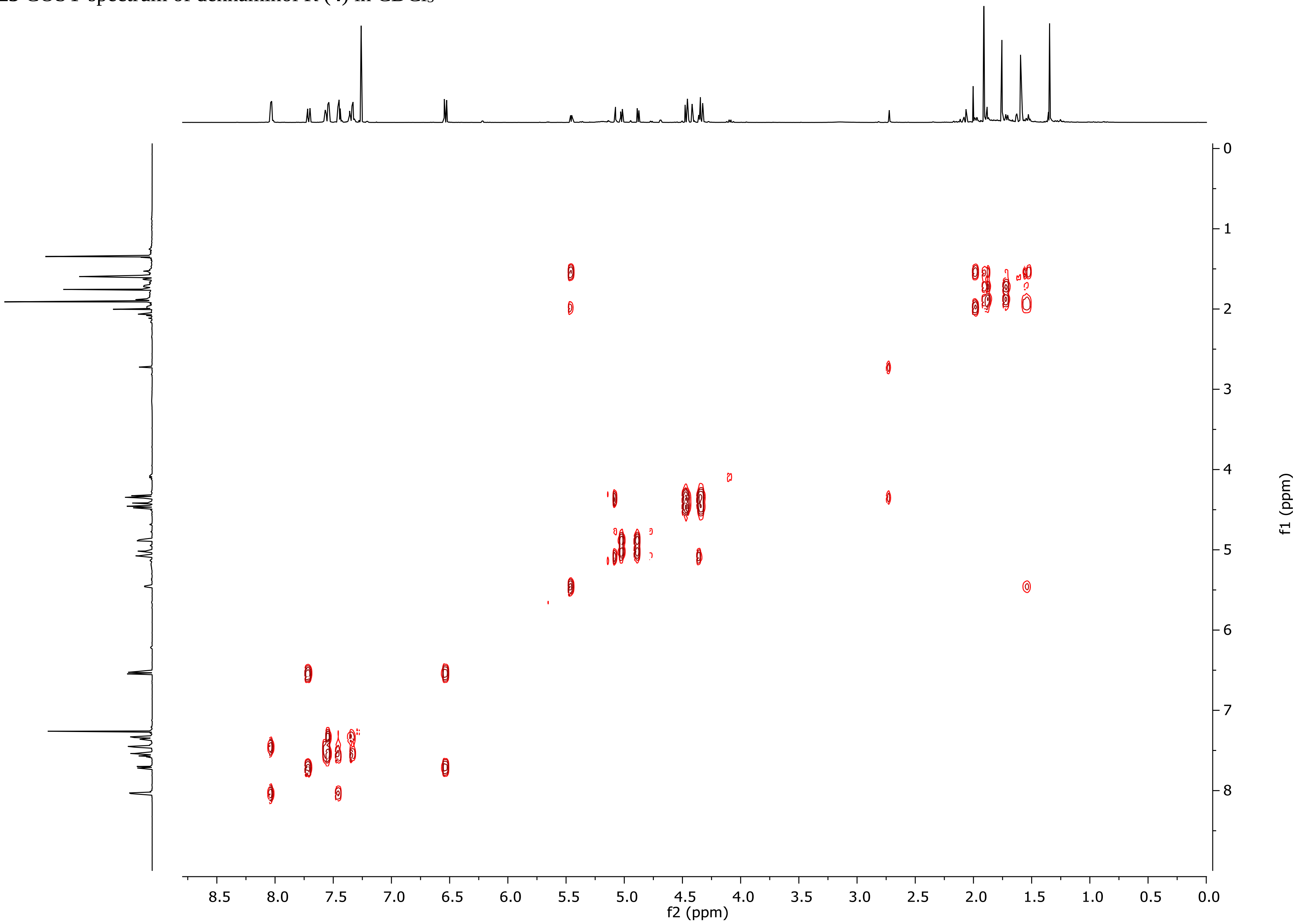

**S24** HSQC spectrum of denhaminol R (**4**) in $CDCl_3$

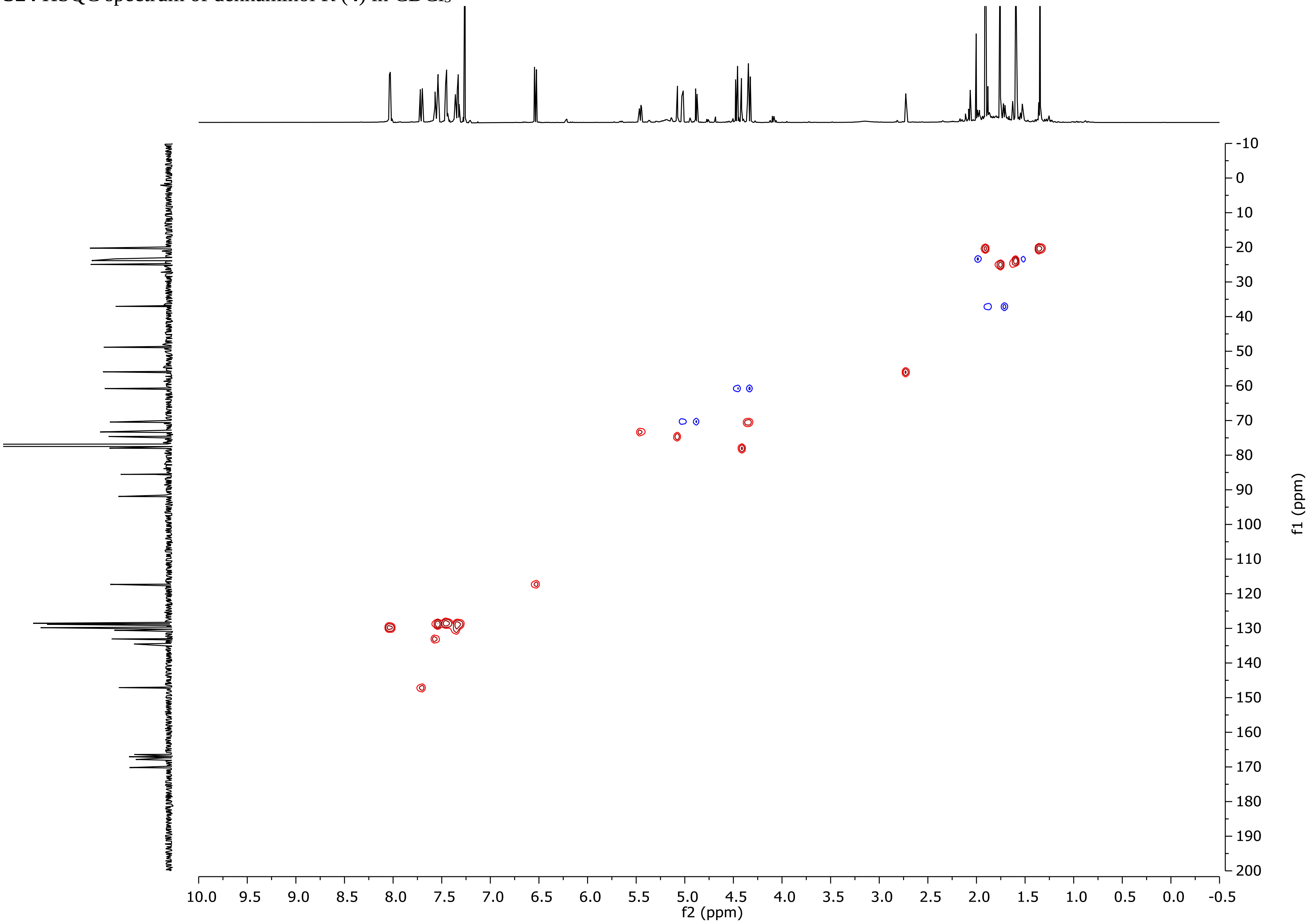

**S25** HMBC spectrum of denhaminol R (**4**) in $CDCl_3$

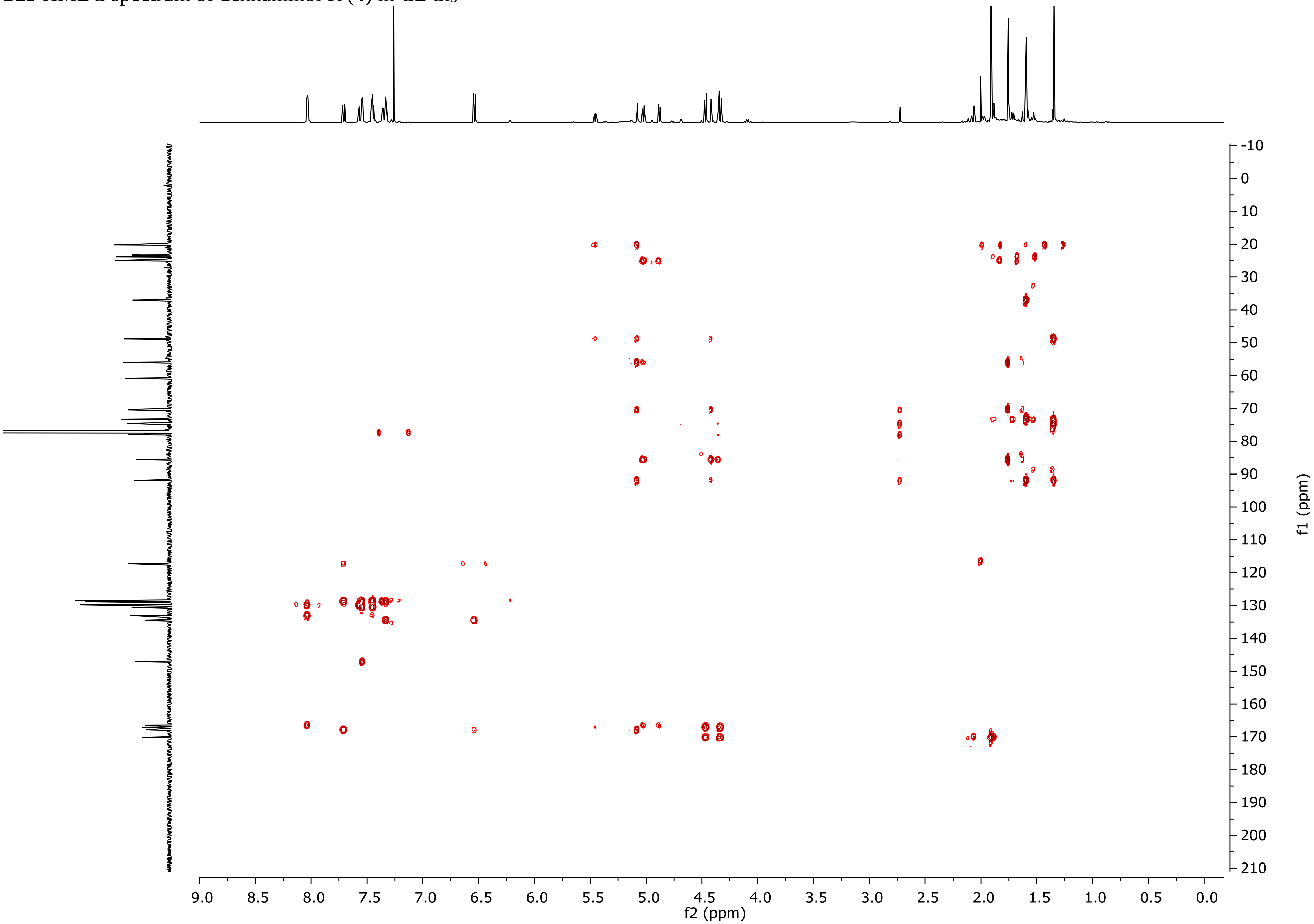

**S26** ROESY spectrum of denhaminol R (**4**) in $CDCl_3$

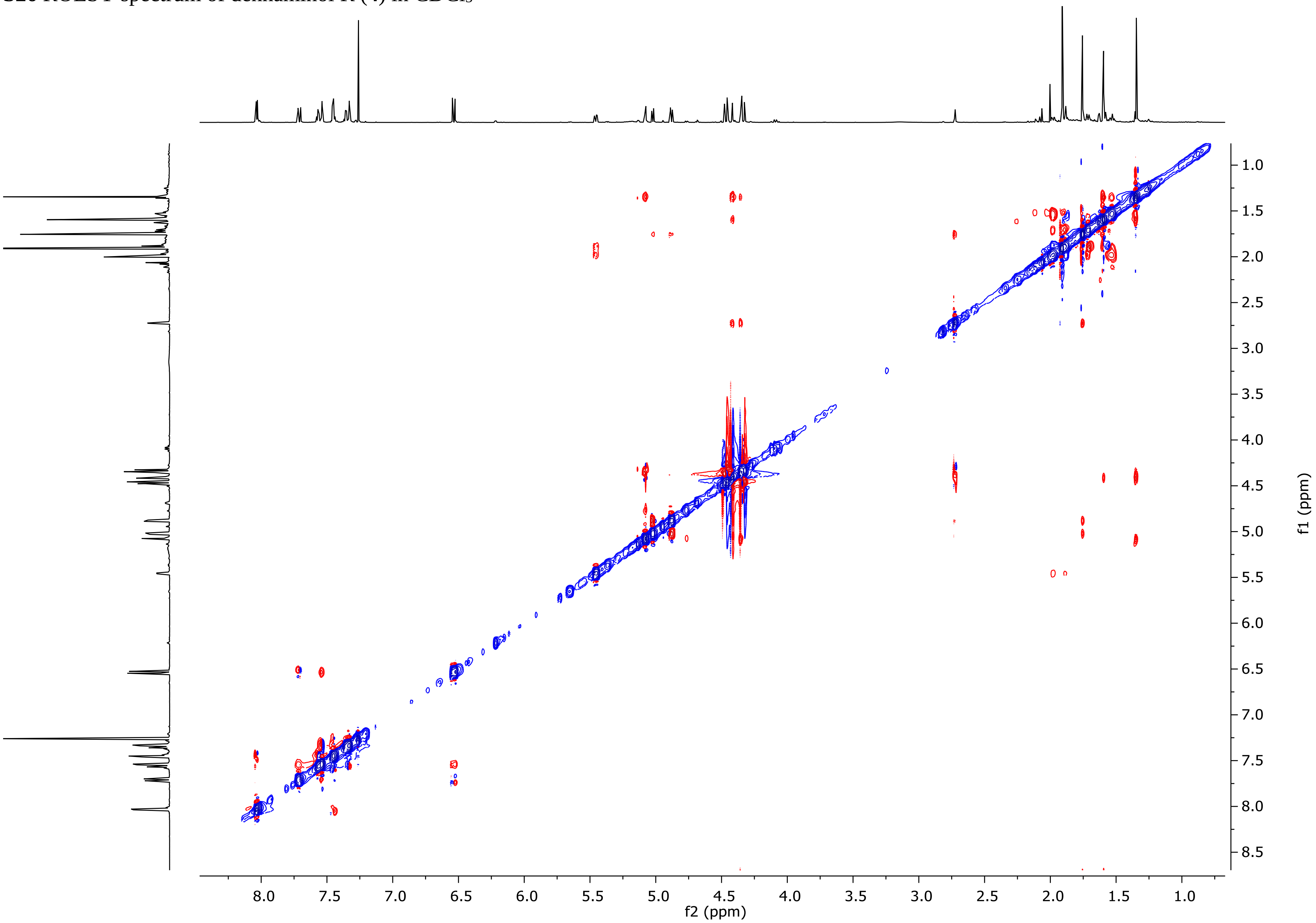

**S27** ECD spectra of denhaminols O–R (**1**–**4**) and denhaminol G (**5**) in MeOH

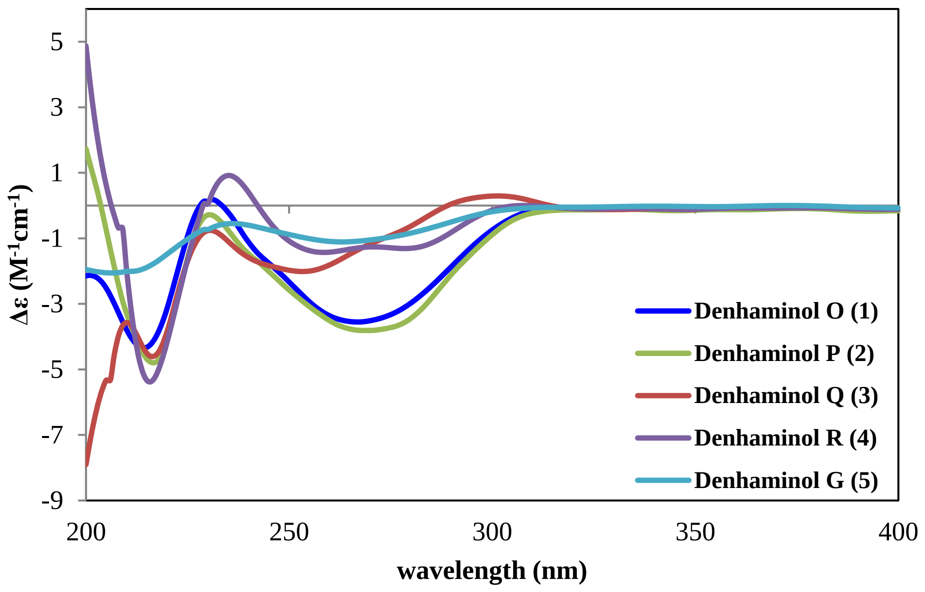


**S28** Diagnostic 2D NMR correlations for denhaminol P (**2**)

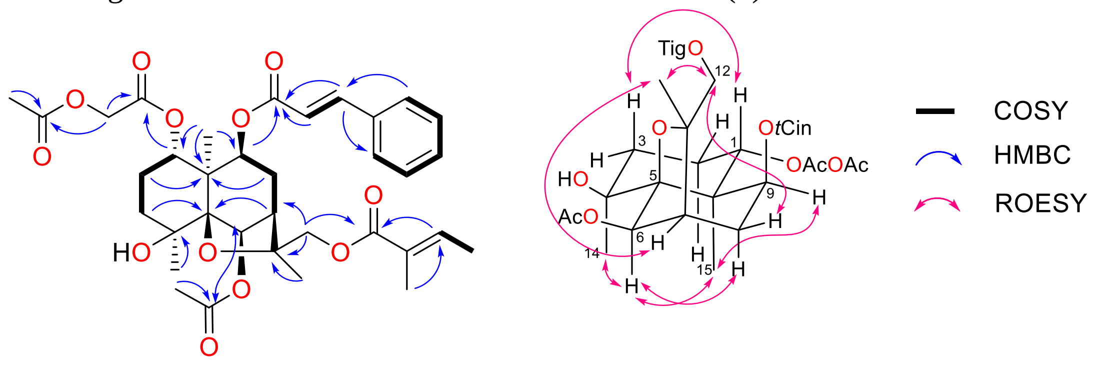

**S29** Diagnostic 2D NMR correlations for denhaminol Q (**3**)

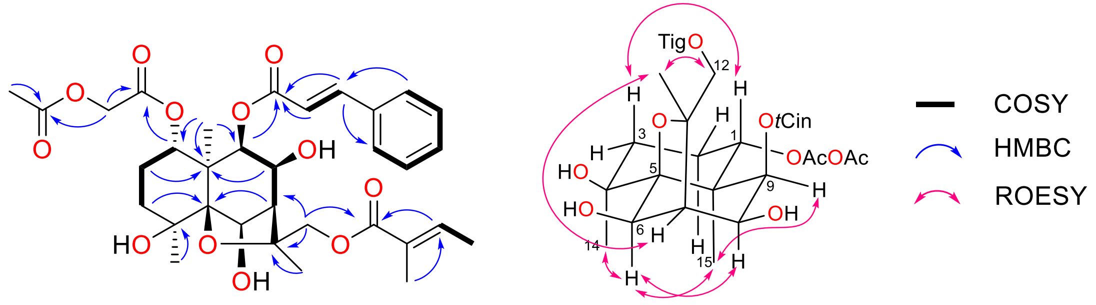


**S30** Diagnostic 2D NMR correlations for denhaminol R (**4**)

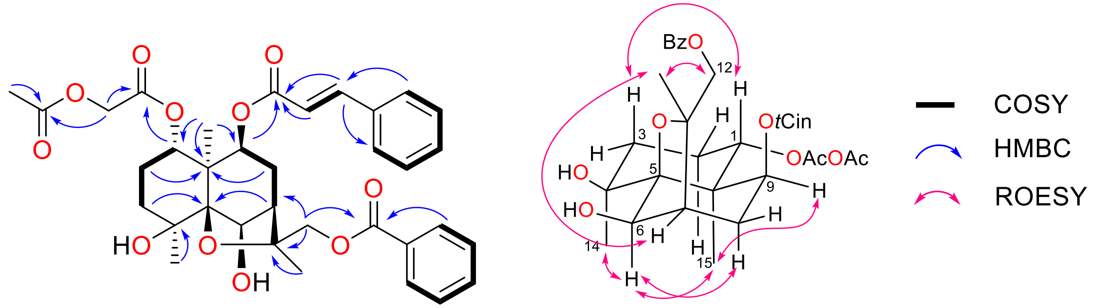

**S31**. Australian Celastraceae plant collection date, location, and voucher specimen codes

| **NatureBank (NB) code** | **Queensland Herbarium (BRI) voucher accession number** | **Sample type** | **Species name** | **Collection date** | **Collection site** |
|---|---|---|---|---|---|
| NB019299 | AQ605014 | Roots | *Denhamia celastroides* (F.Muell.) Jessup | 26 November 1997 | Mt Windsor Tableland, Queensland, Australia |
| NB005745 | AQ603214 | Twigs | *Perrottetia arborescens* (F.Muell.) Loes. | 10 June 1996 | Longlands Gap, Queensland, Australia |
| NB015383 | AQ605014 | Bark | *Denhamia celastroides* (F.Muell.) Jessup | 26 November 1997 | Mt Windsor Tableland, Queensland, Australia |
| NB020738 | AQ605523 | Roots | *Denhamia fasciculiflora* (Jessup) M.P.Simmons | 28 June 1998 | Macrossan Range, Queensland, Australia |
| NB005757 | AQ603214 | Roots | *Perrottetia arborescens* (F.Muell.) Loes. | 10 June 1996 | Longlands Gap, Queensland, Australia |
| NB010702 | AQ602529 | Roots | *Elaeodendron melanocarpum* F.Muell. | 29 December 1995 | Wietalaba, Queensland, Australia |
| NB005647 | AQ603214 | Leaves | *Perrottetia arborescens* (F.Muell.) Loes. | 10 June 1996 | Longlands Gap, Queensland, Australia |
| NB016513 | AQ604981 | Bark | *Elaeodendron melanocarpum* F.Muell. | 26 November 1997 | Mt Windsor Tableland, Queensland, Australia |
| NB003376 | AQ600521 | Mixed | *Hippocratea barbata* F.Muell. | 10 December 1993 | Shiptons Flat, Queensland, Australia |
| NB013676 | AQ604654 | Bark | *Hypsophila halleyana* F.Muell. | 25 October 1997 | Bartle Frere, Queensland, Australia |
| NB004940 | AQ602808 | Bark | *Denhamia cunninghamii* (Hook.) M.P.Simmons | 7 February 1996 | Lappa, Queensland, Australia |
| NB022808 | AQ605663 | Roots | *Denhamia pittosporoides* F.Muell. subsp. *pittosporoides* | 6 November 1998 | Cannondale Scrub, Queensland, Australia |
| NB021847 | AQ605691 | Roots | *Denhamia cunninghamii* (Hook.) M.P.Simmons | 3 November 1998 | Bigge Range, Queensland, Australia |
| NB018975 | AQ605133 | Roots | *Denhamia cunninghamii* (Hook.) M.P.Simmons | 17 March 1998 | Surprise Creek, Queensland, Australia |
| NB001949 | AQ601069 | Mixed | *Hypsophila dielsiana* Loes. | 16 July 1994 | Danbulla, Queensland, Australia |
| NB016128 | AQ605014 | Fruits | *Denhamia celastroides* (F.Muell.) Jessup | 26 November 1997 | Mt Windsor Tableland, Queensland, Australia |
